# Identification of potent SENP1 inhibitors that inactivate SENP1/JAK2/STAT signaling pathway and overcome platinum drug resistance in ovarian cancer

**DOI:** 10.1002/ctm2.649

**Published:** 2021-12-26

**Authors:** Yi Zhang, Huiqiang Wei, Yuan Zhou, Zhuqing Li, Wenfeng Gou, Yunxiao Meng, Wei Zheng, Jing Li, Yiliang Li, Wenge Zhu

**Affiliations:** ^1^ Department of Biochemistry and Molecular Medicine the George Washington University School of Medicine and Health Sciences Washington District of Columbia USA; ^2^ GW Cancer Center the George Washington University Washington District of Columbia USA; ^3^ Tianjin Key Laboratory of Radiation Medicine and Molecular Nuclear Medicine Institute of Radiation Medicine Peking Union Medical College and Chinese Academy of Medical Sciences Tianjin China; ^4^ National Center for Advancing Translational Sciences National Institutes of Health Bethesda Maryland USA

Dear Editor,

Sentrin‐specific protease 1 (SENP1) is a protease that is important for tumor development and drug resistance.^1^, ^2^ Our recent discovery indicates that SENP1 is critical for Janus kinase/signal transducers and activators of transcription (JAK2/STAT) signaling by regulating JAK2 activity through deSUMOylation.^1^ Therefore, targeting SENP1 is a promising approach for cancer therapy and many efforts have been invested into developing SENP1 inhibitors.^3^

Momordin Ιc (Mc) is a natural compound that inhibits SENP1 with higher IC_50_.^4^ To identify potent SENP1 inhibitors, we conducted a drug synergy screening by using a collection of Mc natural compound analogues (Figure 1A). This screening is based on our discovery that overexpressed SENP1 contributes to cisplatin resistance in ovarian cancer (OC) and inhibition of SENP1 by Mc exhibits the great synergistic effects with cisplatin to inhibit resistant OC cancer cells IGROV1 CR.^1^ Thus, if one Mc analogue exhibits synergy with cisplatin in resistant cells, it may target SENP1. The screening identified ursolic acid (UA) and triptolide that re‐sensitized resistant cells to cisplatin with lower IC_50_ (UA: 0.86 μM; triptolide: 1.37 μM) (Figure 1B), suggesting a good synergy with cisplatin. Interestingly, triptolide was reported to inhibit SENP1,^5^ further supporting the feasibility of the screening strategy.

**FIGURE 1:**
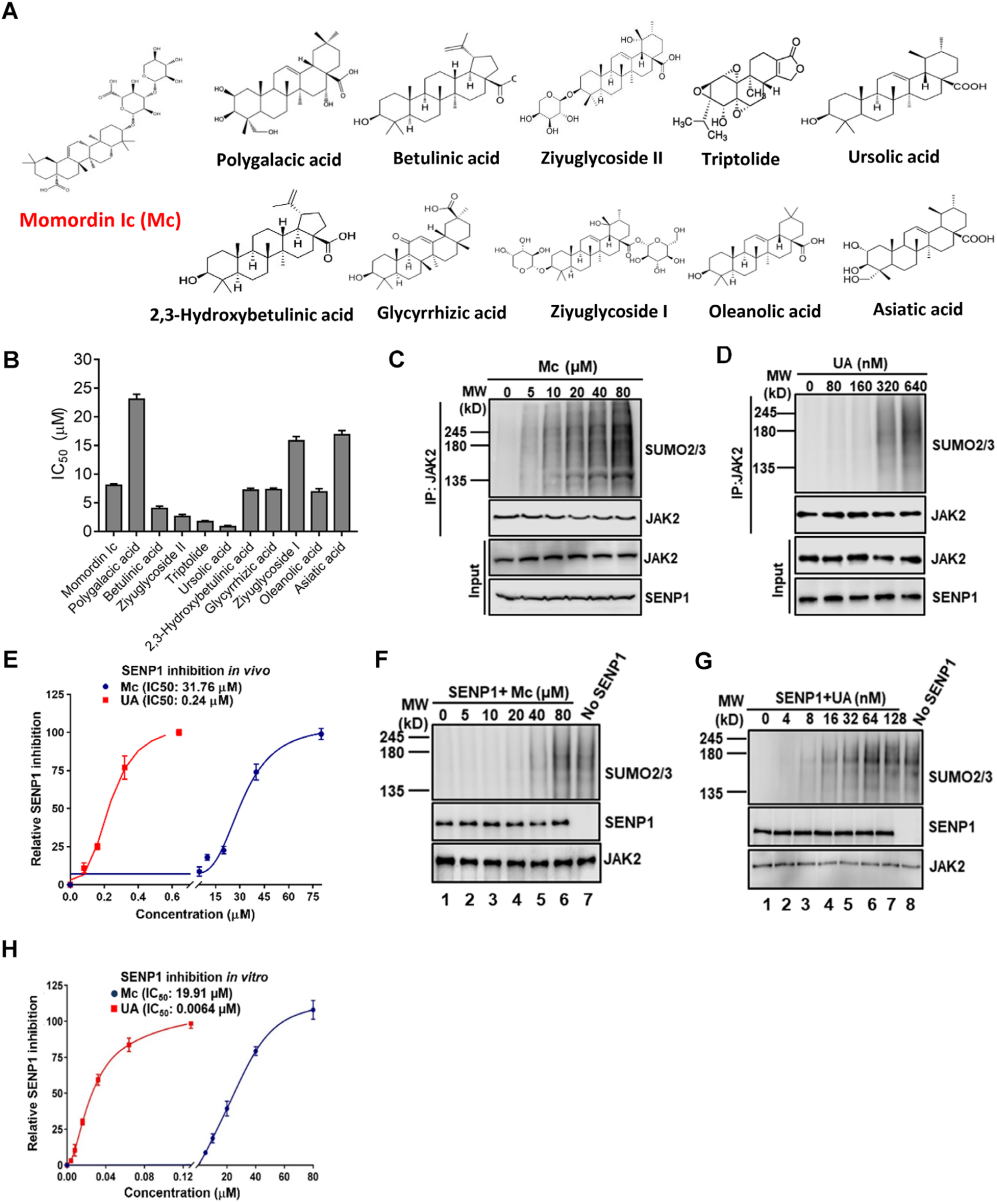
Drug synergy screening to identify potent Sentrin‐specific protease 1 (SENP1) inhibitor ursolic acid. (A) Collection of Momordin Ic (Mc) natural compound analogues. (B) IC_50_ of Mc analogues combined with 2 μM cisplatin to treat IGROV1 CR cells. Cell proliferation was determined by the sulforhodamine B (SRB) assay. (C) Mc inhibits SENP1 activity in cells. IGROV1 CR cells were incubated with Mc at indicated concentrations for 48 h and then harvested for Western blot for indicated proteins. (D) UA inhibits SENP1 activity in cells. IGROV1 CR cells were incubated with UA and then treated as in C. (E) Quantification of SENP1 inhibition by Mc and UA shown in (C) and (D). Intensity of SUMOylated JAK2 was measured by Quantity One software. (F) Mc inhibits SENP1 activity in vitro. Recombinant His‐JAK2 was SUMOylated by in vitro SUMOylation assay, and then SUMOylated JAK2 proteins were incubated with or without recombinant GST‐SENP1 together with Mc for 3 h. (G) UA inhibits SENP1 activity in vitro. In vitro assay was performed as in (F). (H) Quantification of SENP1 inhibition by Mc and UA in vitro *is* shown in (F) and (G). Intensity of SUMOylated JAK2 was measured by Quantity One software

We next examined JAK2 SUMOylation in cells treated with Mc or UA. JAK2 SUMOylation levels were significantly increased upon treatments, indicating that UA and Mc inhibit SENP1 (Figure 1C,D). The IC_50_ of UA (0.24 μM) is much lower than Mc (31.76 μM) (Figure 1E). In vitro deSUMOylation assay confirmed the inhibition of UA on SENP1 with IC_50_ at 0.0064 μM, compared to 19.91 μM by Mc (Figure 1F–H). Cellular thermal shift assay, which is used to compare the thermal stabilization of a protein in ligand‐free and ligand‐bound states,^6^, ^7^ showed that at higher temperatures SENP1 protein was stabilized in cells treated with UA (Figure 2A), indicating a direct interaction between SENP1 and UA. Consistently, UA also stabilized recombinant SENP1 proteins in vitro at higher temperatures (Figure 2B).

**FIGURE 2:**
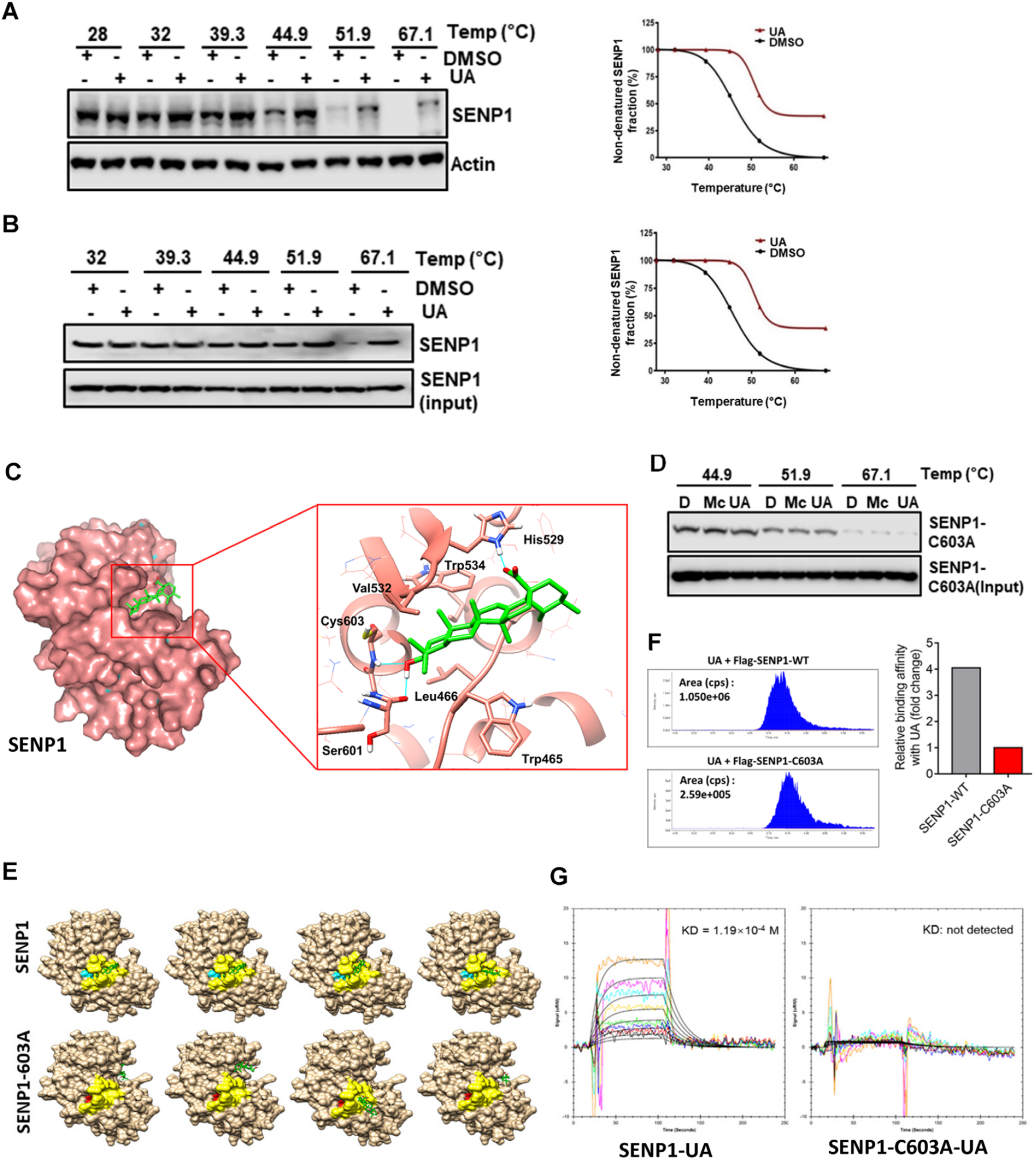
UA directly interacts with Sentrin‐specific protease 1 (SENP1). (A) Cellular thermal shift assay to examine the interaction of UA with SENP1. IGROV1 CR cells pretreated with MG132 (10 μM, 1 h) were incubated with DMSO or UA for 4 h before being shifted to indicated temperatures. SENP1 stability was analyzed by using Western blot against SENP1 antibody. Right panel, quantification of non‐denatured SENP1 fraction shown in the left panel. (B) Cell‐free thermal shift assay to examine interactions of UA with SENP1. Purified SENP1 proteins were incubated with DMSO or UA for 4 h before being shifted to the indicated temperatures. SENP1 stability was analyzed by using Western blot against SENP1 antibody. Right panel, quantification of non‐denatured SENP1 fraction shown in the left panel. (C) The predicted docking pose of UA (C: green; H: white; O: red) at the hydrophobic catalytic cleft of SENP1 (C: salmon; H: white; O: red; N: blue). Key amino acid residues were highlighted. H‐bonds were shown in sky‐blue lines. The pictures were generated by Chimera. (D) In vitro thermal shift assay to analyze the interactions of UA and Mc with purified mutant GST‐SENP1 (C603A). Stability of SENP1(C603A) was analyzed by using Western blot against SENP1 antibody. (E) Docking poses of UA (C: green; H: white; O: red) with SENP1 and SENP1‐C603A. Surface of the catalytic cleft was shown in yellow; the surfaces of Cys603 and Ala603 were shown in cyan and red, respectively. The pictures were generated by Chimera. (F) Mass‐spectrometry to measure the amount of UA associated with IPs of wild‐type SENP1 and SENP1‐C603A proteins. Left; Ultra‐high performance liquid‐chromatography‐MS/MS analyses of associated UA in indicated samples, right; quantification of data shown in left (the binding affinity of UA with SENP1‐C603A was normalized as 1). (G) Surface plasmon resonance (SPR) to characterize the interactions of UA with purified recombinant SENP1 or SENP1‐C603A proteins

The molecular docking showed that UA entered the hydrophobic catalytic cleft of the SENP1 (Figure 2C). The 3‐hydroxyl group of UA forms two hydrogen bonds with key catalytic residues Cys603 and Ser601. The 28‐carboxyl group was located at the opening of SENP1 and formed an extra hydrogen bond with His529. Hydrophobic residues Trp465, Leu466, Ile471, Val532 and Trp534 formed a hydrophobic cleft, which interacts with pentacyclic triterpenoid scaffold (rings A, B and C).

Cys603 is a key catalytic amino acid in hydrolyzing SUMO tags of substrate proteins and forms a hydrogen bond with UA^8^ (Figure 2C), suggesting Cys603 may be critical for UA‐SENP1 interaction. Indeed, the thermal stability of purified mutant SENP1(C603A) was not increased after the addition of Mc and UA compared to dimethyl sulfoxide (DMSO) treatment (Figure 2D), indicating that C603 is critical for interactions of SENP1 with UA and Mc. Strikingly, molecular docking indicated that C603A mutation resulted in alteration of the hydrophobic pocket structure of SENP1, preventing UA from entering the catalytic cleft of SENP1(C603A) (Figure 2E). Consistently, both mass‐spec and surface plasmon resonance analyses indicated that SENP1(C603A) exhibited decreased binding affinities with UA compared to SENP1 (Figure 2F,G and Table S1).

The disadvantages such as poor water solubility, fast metabolism and low bioavailability limited UA's clinical application.^9^ We, therefore, developed several new UA derivatives by rational structure modification to improve its druggability. Since the 3‐hydroxyl group directly interacted with Cys603, while the 28‐carboxyl group is located at the opening of SENP1 and pointed to the solvent region, we decided to modify the 28‐carboxyl group and retain the 3‐hydroxyl group. To this end, eight derivatives (UAMMC1‐7&9) were synthesized from UA by introducing various hydrophilic groups to a 28‐carboxyl position (Figure 3A). QikProp module predicted the better pharmacokinetic properties of these derivatives than UA (Table S2). The water solubilities of UA derivatives were greatly improved (Table S3). Strikingly, UAMMC9 exhibited the best water solubility (39.7 μg/ml).

**FIGURE 3:**
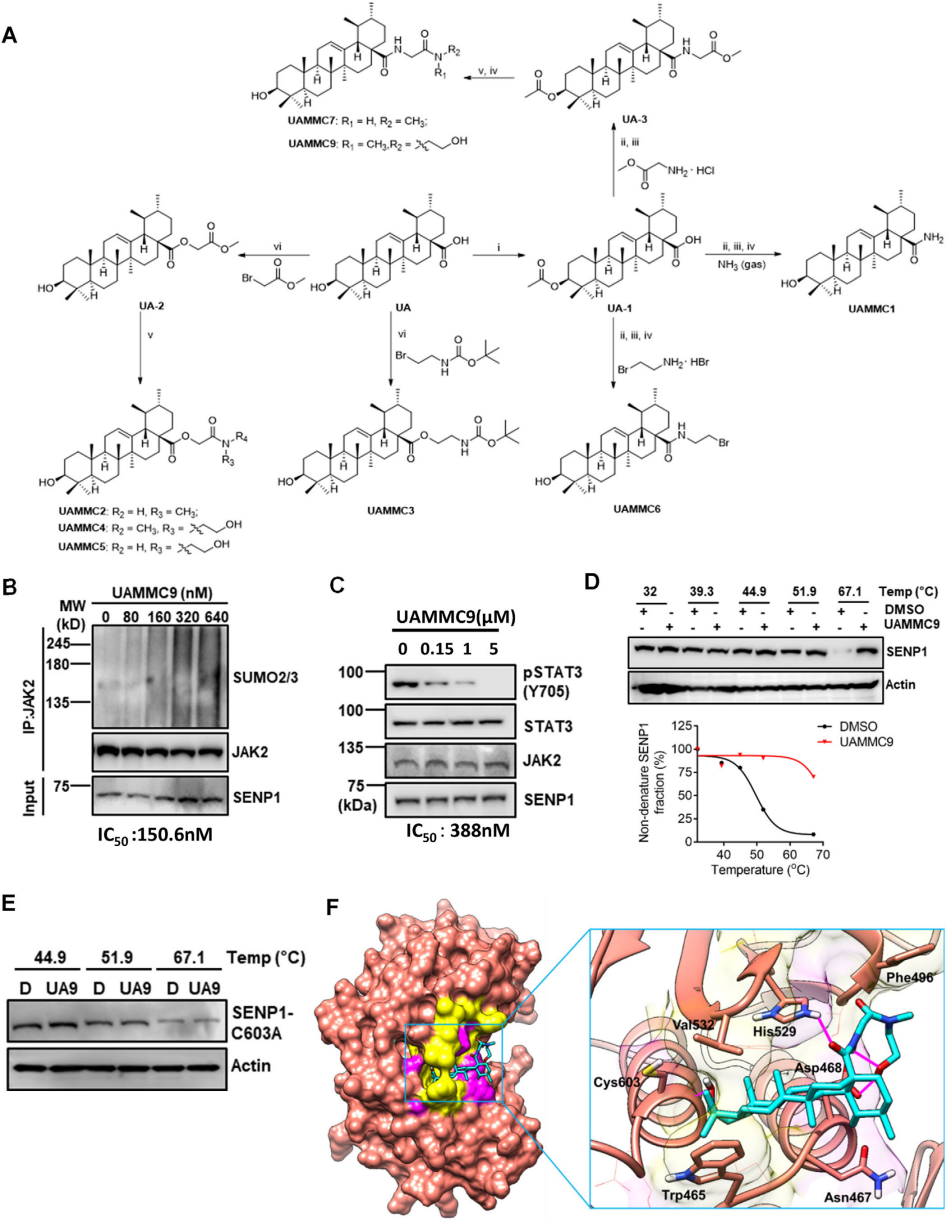
Identification of UAMMC9 from UA derivatives. (A) Synthesis of UA derivatives. Reagents and conditions: (i) Ac_2_O, DMAP, Pyridine, r.t.; (ii) Oxalyl chloride, CH_2_Cl_2_, r.t., overnight; (iii) amines, TEA, r.t., 0.5–4 h; (iv) NaOH a.q., MeOH/THF, r.t.; (v) amines, MeOH, reflux; (vi) K_2_CO_3_, DMF, 60°C. The detail of synthesis was included in supplemental materials. (B) UAMMC9 inhibits Sentrin‐specific protease 1 (SENP1) activity in cells. IGROV1 CR cells were incubated with UAMMC9 at indicated concentrations for 48 h, followed by treatment as shown in Figure 1C. (C) IGROV1 CR cells treated with indicated UAMMC9 for 48 h. Cells were then harvested and lysates were resolved on sodium dodecyl sulphate‐polyacrylamide gel electrophoresis, followed by immunoblotting for indicated proteins. (D) Cellular thermal shift assay to examine the interaction of UAMMC9 with SENP1. IGROV1 CR cells were incubated with DMSO or UAMMC9 for 4 h before treatment as in Figure 2A. (E) In vitro thermal shift assay to analyze the interactions of UAMMC9 with purified GST‐SENP1(C603A). Stability of SENP1(C603A) was analyzed by using Western blot against SENP1 antibody. (F) The predicted docking pose of UAMMC9 (C: cyan; H: white; O: red; N: blue) at the hydrophobic catalytic cleft of SENP1 (C: salmon; H: white; O: red; N: blue). Surface of hydrophobic amino acid residues in catalytic cleft was shown in yellow and that of hydrophilic amino acid residues was shown in magenta. Key amino acid residues were highlighted. H‐bonds were shown in magenta lines. The pictures were generated by Chimera.

The synergy screen identified compounds UAMMC3, 5 and 9 with lower IC_50_ to inhibit resistant cells together with cisplatin (Figure S1A). However, UAMMC3 and 5 alone exhibited higher toxicity to cells (Figure S1B), we, therefore, excluded these two compounds and focussed on UAMMC9. Strikingly, UAMMC9 significantly inhibited SENP1 activity in cells with IC_50_ at 150 nM (Figure 3B), and in vitro with IC_50_ at 195.7 nM (Figure S2A,B). Given SENP1 activates JAK2/STAT3 signaling by deSUMOylating JAK2,^1^ it was expected that UAMMC9 significantly reduced the phosphorylation levels of STAT3 at Y703 (Figure 3C and Figure S2E). UAMMC9 directly interacted with SENP1 as indicated by thermal shift assays (Figures 3D and Figure S2C,D), and C603 is also critical for SENP1‐UAMMC9 interaction (Figure 3E). The molecular docking indicated that UAMMC9 entered the catalytic cleft of SENP1 in a similar manner to UA (Figure 3F).

UA and UAMMC9 exhibited great synergy with cisplatin to inhibit IGROV1 CR cells (Figures 4A,B and Figure S3A).  To evaluate the therapeutic potential of UA and UAMMC9 with cisplatin in vivo, we subcutaneously implanted IGROV1 CR cells into nude mice to form drug‐resistant tumors, which, then, were treated with UA or UAMMC9, cisplatin, or the combination of both via intraperitoneal injection. The combinational treatments with cisplatin and UA or UAMMC9 exhibited a remarkable synergy to reduce tumor growth as compared to UA, UAMMC9 or cisplatin alone (Figure 4C–F). UAMMC9 could inhibit the activation of STAT3 in vivo (Figure 4G). Importantly, the treatments had minor toxicity (Figures S3B–D). Strikingly, UAMMC9 exhibited a similar inhibition on tumors as UA even though it was used 5‐fold less than UA, demonstrating a greatly improved druggability.

**FIGURE 4:**
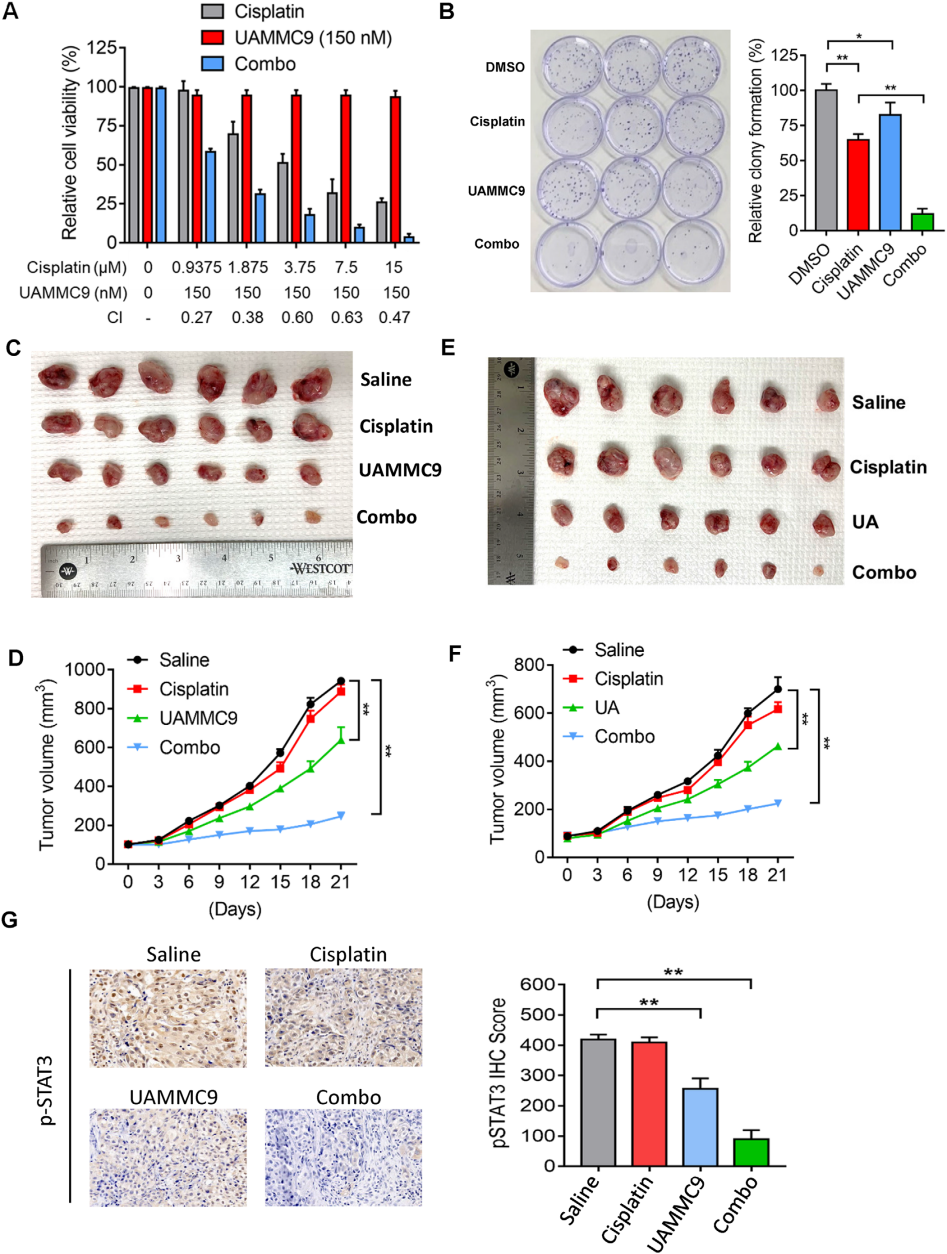
Inhibition of Sentrin‐specific protease 1 (SENP1) by UA and UAMMC9 overcomes cisplatin resistance in ovarian cancer. (A) The synergistic effects of cisplatin and UAMMC9 in IGROV1 CR cells. Cell viability was measured by SRB assay. Concentrations of cisplatin and UAMMC9 as well as the combination index (CI) were indicated. Data were represented as mean ± SD (*n*  =  3). (B)  Colony formation assay in cells treated as indicated. Data were represented as mean ± SD (*n*  =  3). **p < 0.01; *p < 0.1 (C) Photograph of tumors from IGROV1 CR xenograft mice treated with control, cisplatin (8 mg/kg/2 days intraperitoneally), UAMMC9 (2 mg/kg/2 days), and UAMMC9 + cisplatin (2 mg/kg/2 days of UAMMC9 + 8 mg/kg/2 days of cisplatin). (D) Growth curves of xenograft tumors are shown in (C). Data are represented as means ± SEM, *n*  =  6 mice/group. **p  <  0.01. (E) Photograph of tumors from IGROV1 CR xenograft mice treated with control, cisplatin (8 mg/kg/2 days intraperitoneally), UA (10 mg/kg/2 days), and UA + cisplatin (10 mg/kg/2 days of UA + 8 mg/kg/2 days). (F) IGROV1 CR xenograft tumor growth curve by indicated treatments. Data are represented as means ± SD, *n* = 6 tumors/group. **p < 0.01. (G) Immunohistochemistry (IHC) staining against pSTAT3 antibodies of tumor samples from four groups of mice (magnification, 200x) (Upper). Right panel, quantification of pSTAT3 IHC score in tumor samples from mice treated with indicated drugs. **p < 0.01

In summary, we identified UA and UAMMC9 that directly bind to SENP1 catalytic site to inhibit its activity at the nM levels and overcome OC platinum resistance. UA is currently in clinical trials to treat metabolic syndrome and sarcopenia and has demonstrated its safety.^10^ Thus, this study provides not only the evidence supporting a potential clinical trial to treat platinum‐resistant OC and SENP1‐dependent cancers, but also a new SENP1 inhibitor UAMMC9 with excellent druggability for further developing anti‐cancer drugs by targeting SENP1/JAK2/STAT signaling pathway.

## CONFLICT OF INTEREST

The authors declare that they have no conflict of interest.

## Supporting information



Supporting Information
