# Supplementary material for: Identification of potent SENP1 inhibitors that inactivate SENP1/JAK2/STAT signaling pathway and overcome platinum drug resistance in ovarian cancer
Source: Clin Transl Med. 2021 Dec 26;11(12):e649. doi: 10.1002/ctm2.649 (PMC8710297; doi:10.1002/ctm2.649)
Supplement: Supplementary file 1 — Supporting Information [file CTM2-11-e649-s001.pdf]

Supplemental materials and motheds

Identification of potent SENP1 inhibitors that inactivate SENP1/JAK2/STAT signaling pathway and overcome platinum drug resistance in ovarian cancer

Yi Zhang, Huiqiang Wei, Yuan Zhou, Zhuqing Li, Wenfeng Gou, Yunxiao Meng, Wei Zheng, Jing Li, Yiliang Li, Wenge Zhu

This file includes:

Supplementary Figures S1-S3

Supplementary Table. S1-S3

Supplementary Materials and Methods

A

Cisplatin + UAMMC	IC50 (μM)
DMSO	14.22
1	8.345
2	13.92
3	0.080
4	12.74
5	4.482
6	14.47
7	15.35
9	3.396

B

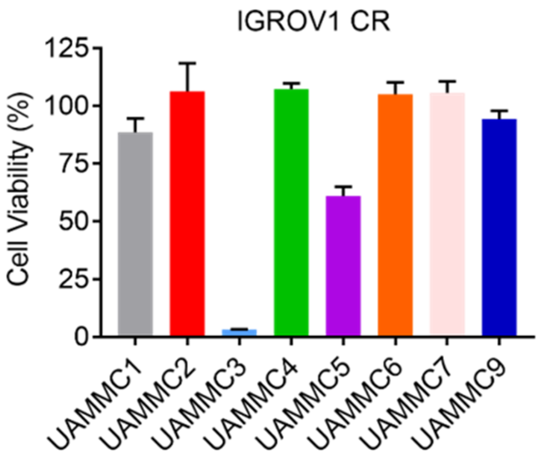

Figue S1. (**A**) IC_{50} of UA derivatives combined with 2 μM cisplatin to treat IGROV1 CR cells. Cell proliferation was determined by SRB assay. (**B**) Viability of IGROV1 CR cells treated by indicated compounds for 48 hrs. SRB assay was used to determine cell viability.

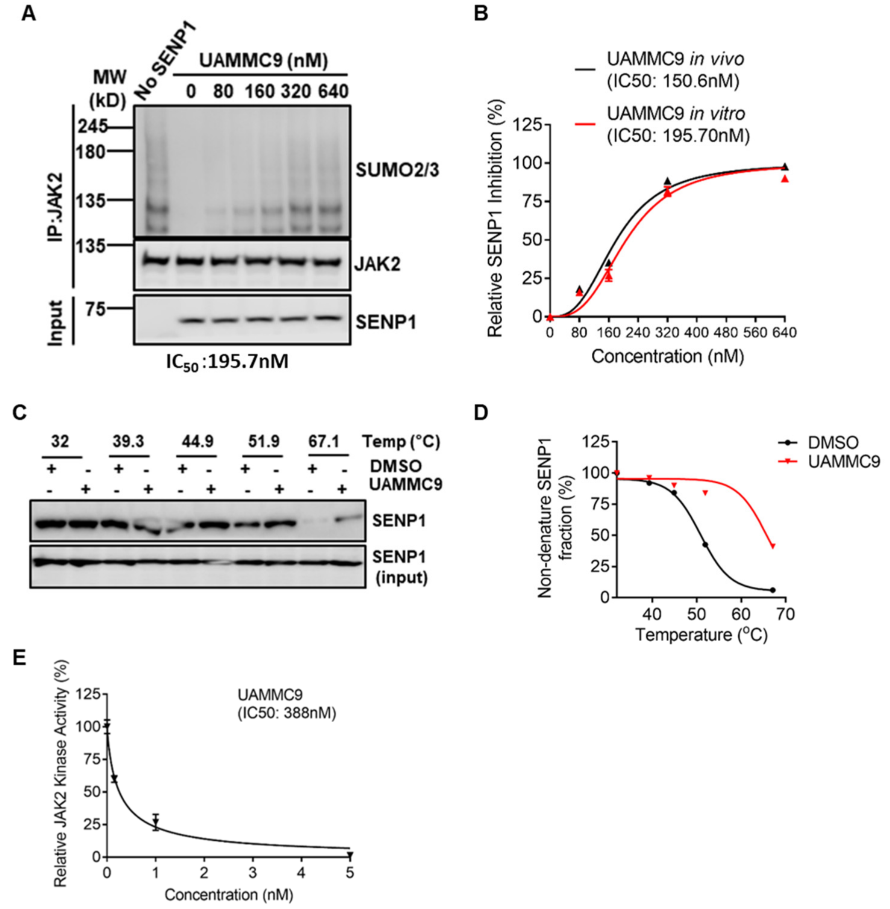

Figue S2. (**A**) UAMMC9 inhibits SENP1 activity *in vitro*. The assay was performed as in Figure 1F. (**B**) Quantification of SENP1 inhibition by UAMMC9 *in vitro and in vivo* shown in A and Figure 3B . Intensity of SUMOylated JAK2 in was measured by Quantity One software.(**C**) Cell free thermal shift assay to examine interactions of UAMMC9 with SENP1. Purified SENP1 proteins were incubated with DMSO or UA for 4 hrs before shifted to the indicated temperatures. SENP1 stability was analyzed by using Western blot against SENP1 antibody. (**D**) Quantification of non-denatured SENP1 fraction shown in C. (**E**) Quantification of the levels of pSTAT3 as shown in Figue 3C.

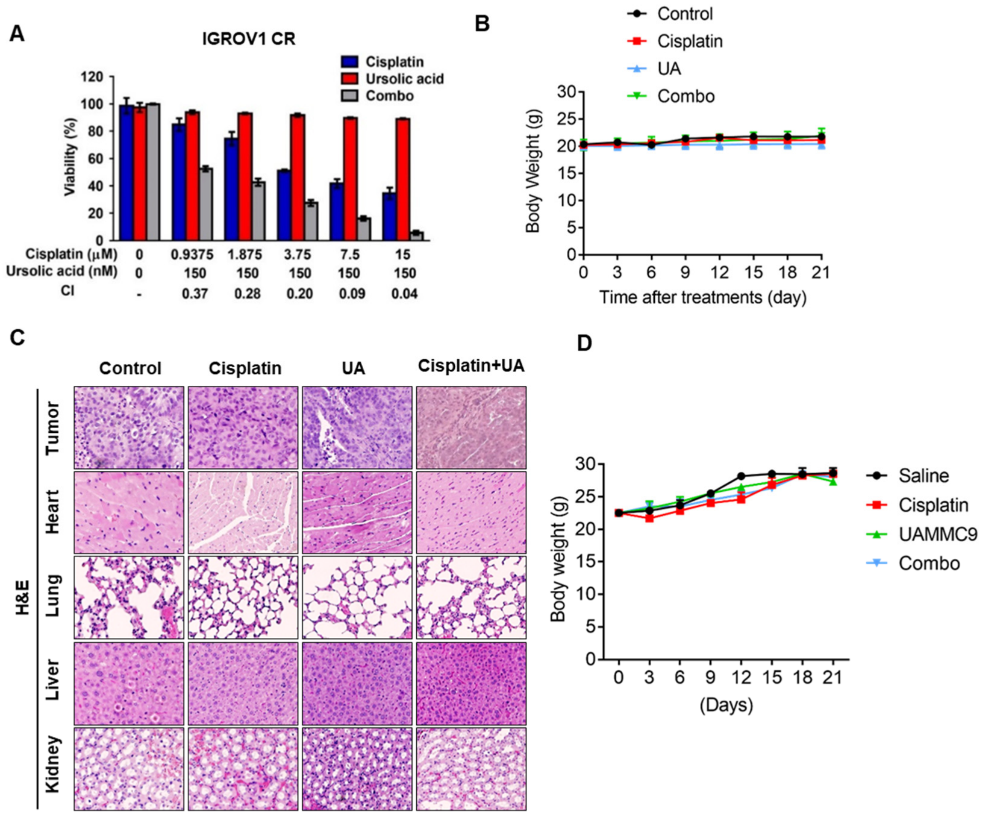

Figue S3. (**A**) The synergistic effects of cisplatin and ursolic acid in IGROV1 CR cells. Concentrations of cisplatin and ursolic acid as well as the CI index were indicated. Data were represented as mean ± SD (n = 3). (**B**) Body weight changes of mice in each group treated with control, cisplatin (8mg/kg/2day intraperitoneally), UA (10mg/kg/2days), and UA+cisplatin (10mg/kg/2day of UA +8mg/kg/2day). Data are represented as means ± SD, n=6 tumors/group. (**C**) H&E staining of paraffin-embedded 3-μm-thick tissue sections of the tumor, heart, lung, liver and kidney from 4 groups of mice (magnification, ×200) treated in B. Control: normal tubular structure with thin interstitial tissue and capillary. Cisplatin: slightly dilated capillaries can be seen in the interstitial between the tubular. UA: tubular epithelium cells become slightly shorter. Cisplatin +UA: slightly dilated capillaries can be seen in the interstitial between the tubular and tubular epithelium cells become slightly shorter. (**D**) Body weight changes of mice in each group shown in Figure 4D. Data are represented as means ± SD, n=6 tumors/group.

Table S1. Binding affinities of dominant docking poses with SENP1-wild and SENP1-C603A

UA-SENP1-wild				UA-SENP1-C603A			
Mode	Affinity (kcal/mol)	Dist from best mode		Mode	Affinity (kcal/mol)	Dist from best mode	
		rmsd l.b.	rmsd u.b.			rmsd l.b.	rmsd u.b.
1	-9.0	0.000	0.000	1	-7.5	12.634	19.359
2	-8.0	1.375	2.505	2	-7.3	11.896	14.894
3	-8.0	1.524	2.776	3	-7.1	12.689	23.873
4	-7.3	2.399	5.658	4	-6.4	13.031	19.580

Table S2. Prediction of pharmacokinetic properties of UA and its derivatives

molecule	UA	UAMMC1	UAMMC2	UAMMC3	UAMMC4	UAMMC5	UAMMC6	UAMMC7	UAMMC9
CNS	-1	-1	-1	-1	-2	-2	0	-1	-2
mol_MW	456.707	455.723	527.786	599.893	571.839	557.812	562.672	526.801	570.854
dipole	4.192	6.38	8.099	3.604	4.502	3.042	7.332	4.73	7.585
SASA	691.331	694.556	818.653	873.297	915.338	853.335	778.822	785.971	845.203
FOSA	577.663	575.94	689.884	759.832	770.838	691.875	627.881	671.946	703.18
FISA	100.321	105.099	115.619	100.199	131.613	148.196	58.267	100.8	128.867
PISA	13.348	13.517	13.15	13.267	12.887	13.264	13.266	13.225	13.156
WPSA	0	0	0	0	0	0	79.407	0	0
volume	1397.751	1404.054	1639.501	1823.029	1820.268	1717.704	1569.185	1603.574	1734.223
donorHB	2	3	2	2	2	3	2	2.25	2.25
accptHB	3.7	4.2	6.2	6.2	8.4	7.9	4.2	5.95	8.15
dip^2/V	0.012569	0.028987	0.040005	0.007126	0.011135	0.005387	0.034263	0.013949	0.033178
ACxDN^.5/SA	0.007569	0.010474	0.01071	0.01004	0.012978	0.016035	0.007627	0.011355	0.014464
glob	0.874511	0.873066	0.821371	0.826416	0.787663	0.812851	0.838513	0.842981	0.825925
QPpolrz	48.627	48.881	57.016	63.078	62.321	58.22	54.203	55.579	58.881
QPlogPC16	12.361	12.676	14.635	16.016	16.525	15.803	14.123	14.191	15.551
QPlogPoct	21.128	23.187	25.688	27.341	28.226	28.065	23.701	25.068	27.702
QPlogPw	8.289	12.084	13.444	10.305	13.471	15.216	9.387	13.278	14.594
QPlogPo/w	6.185	4.638	5.437	7.337	5.534	4.83	6.515	4.755	4.479
QPlogS	-7.008	-5.893	-6.882	-8.743	-8.113	-6.743	-7.941	-6.405	-6.432
CIQPlogS	-6.947	-5.958	-6.783	-8.906	-6.8	-6.62	-8.303	-6.194	-6.219
QPlogHERG	-1.819	-2.276	-3.111	-4.404	-3.655	-3.249	-2.834	-1.387	-1.663
QPPCaco	280.646	639.651	403.999	1111.002	446.913	254.784	2115.995	573.343	353.518
QPlogBB	-0.432	-0.476	-0.82	-0.776	-1.273	-1.354	-0.035	-0.623	-1.118
QPPMDCK	159.336	493.786	385.217	554.322	264.1	178.562	4060.253	546.518	281.779
QPlogKp	-3.129	-3.217	-3.22	-2.743	-3.228	-3.532	-2.163	-2.947	-3.176
IP(eV)	9.388	9.293	9.467	9.399	9.264	9.38	9.394	9.198	9.107
EA(eV)	-1.108	-1.212	-0.8	-0.809	-0.805	-0.878	0.452	-0.937	-0.831
#metab	3	4	4	3	5	5	3	4	5
QPlogKhsa	1.399	1.064	1.322	2.063	1.23	1.013	1.519	0.945	0.743
HumanOralAbsorption	1	3	1	1	1	1	1	1	1
PercentHumanOralAbsorption	94.019	100	79.511	100	80.867	85.334	100	91.198	85.826
SAfluorine	0	0	0	0	0	0	0	0	0
SAamideO	0	24.285	36.822	0	12.26	23.164	14.804	35.377	28.321
PSA	62.497	65.981	88.344	90.262	98.882	110.573	50.435	84.654	98.903
#NandO	3	3	5	6	6	6	3	5	6
RuleOfFive	1	0	2	2	2	1	2	1	1
#ringatoms	22	22	22	22	22	22	22	22	22
#in34	0	0	0	0	0	0	0	0	0
#in56	22	22	22	22	22	22	22	22	22
#noncon	20	20	20	20	20	20	20	20	20
#nonHatm	33	33	38	43	41	40	36	38	41
RuleOfThree	1	1	1	1	1	1	1	1	1
Jm	0	0	0	0	0	0	0	0	0

Table S3. Water solubilities of UA and its derivatives

Compound	UA	UAMMC4	UAMMC5	UAMMC7	UAMMC9
Water solubility （μg/mL）	<0.5	2.89±0.02	14.6±0.36	4.98±0.07	39.7±0.28

Materials and Methods

1. Antibodies and reagents

Antibodies used for Western blot, IHC, and IF are: Anti-SENP1 (ab108981; Abcam), JAK2 (3230S; Cell Signaling Technology), SUMO2/3 (ab3742; Abcam), STAT3 (ab5073; Abcam), p-STAT3 (ab76315; Abcam), Bcl-xL (2764S; Cell Signaling Technology), and β-actin (5441; Sigma-Aldrich).

Cisplatin (1134357; Sigma) was dissolved in sterile saline for cell survival and xenograft studies. Momordin Ic (A14773; Adooq Bioscience) was dissolved in DMSO. Ursolic acid (A10961; Adooq Bioscience) was dissolved in DMSO. Collection of Mc natural compound analogs were purchased from Sigma. Detailed procedure to synthesize UA derivatives was included in supplemental material and methods.

2. Cell culture and resistance cell line establishment

Cisplatin resistant ovarian cancer IGROV1 CR cells were described as previously (1,2). Cells were cultured at 37 °C in DMEM with 10% Fetal Bovine Serum (FBS). All the cells were cultured at in a humidified incubator with 5% CO_2 atmosphere.

3. *In vitro* SUMOylation and de-SUMOylation assays

The SUMOylation reactions were performed according to the manufacture's instruction (BML-UW8955-0001; ENZO) and were described previously (1). Briefly, 200 nM of purified HIS-JAK2 was mixed with recombinant SUMOs in reaction buffer (20 µl) containing Mg-ATP, SUMO E1, and SUMO E2, followed by incubation at 37°C for 60 min. For deSUMOylation assay, SENP1 was added to above reaction 60 min after SUMOylation reaction, followed by incubation for additional 60 min. The reactions were then subjected to Western blot to evaluate SUMOylation level.

4. Protein expression and purification

His-tagged or GST-tagged proteins were purified as described previously (1).

5. Cell viability assay

Cell viability was detected using Sulforhodamine B (SRB) assay as described previously (3). Absorbance at 510 nm was detected using a SpectraMax Reader (Molecular Devices) and analyzed with SoftMax Analysis Software (Molecular Devices). Combinational index (CI) values were calculated using CompuSyn software (4).

6. UPLC-MS/MS analysis of interaction between UA and SENP1 proteins

(1) Sample preparation: Flag-SENP1-WT and Flag-SENP1-C603A were transfected into HEK293T cells with lipofectamine2000 as the manufacture described. Then cell lysis was collected and sonicated 10 sec three times. After centrifuge, the supernatant was collected and incubated with Anti-FLAG-M2 Affinity beads overnight. Next, the co-immunoprecipitates were incubated with ursolic acid (5µM) at room temperature for 3hr and collected for the next procedures. 1 mL methanol was added to each sample with beads followed by vibration

at room temperature for 2 h. After centrifugation at 3000×g for 10 min, the supernatant was transferred into a sample vial and dried with Speedvac (Fisher Scientific). Solutions for standards were prepared by using a series of different concentrations of ursolic acid (i.e., 0.1, 0.5, 1.0, 2.0, 5.0 ug/mL in methanol). Each sample was injected for analysis by liquid chromatography-tandem mass spectrometry (UPLC-MS/MS) with multiple reaction monitoring (MRM) mode. (2) UPLC-MS/MS analysis Samples were analyzed with Acquity UPLC system (Waters) coupled with a Q-TRAP 6500 mass spectrometer (Sciex). Specifically, 5 µL of each sample was loaded onto a C18 column (Waters Acquity UPLC BEH C18 1.7µm, 2.1mm x 50 mm). A 15-min gradient of buffer A (water) and buffer B (ACN) was used for separation: 50% buffer B at 0 min, 50% buffer B at 1 min, 90% buffer B at 3 min, 90% buffer B at 12min, 50% buffer B at 12.1min, 50% buffer B at 15min. Data were acquired on the Q-TRAP 6500 mass spectrometer using negative mode with the ion spray voltage of -4.5kV, GS1 75 psi, GS2 80, CUR 30 psi, CAD Medium, and an interface heater temperature of 350°C. Mass spectra were recorded with Analyst TF 1.6.2 software in the MRM mode (Q1 mass: 455 Da, Q3 mass:455.1 Da, Time:100 ms, CE: -20, DP: -50: EP:-7, CXP: -12). (3) Data analysis: Data files were analyzed with quantitated with the "Quantitation Method" in the Analyst software (Analyst TF 1.6.2).

7. Surface Plasmon Resonance (SPR) measurements

Surface Plasmon Resonance (SPR) were performed by using a Reichert 4SPR system (Reichert Technologies, Depew, New York, USA) to measure the interactions of UA with purified recombinant SENP1 or SENP1-C603A proteins. The recombinant SENP1 and SENP1(C603A) dissolved with sodium acetate solution (pH = 4.0) were captured at the concentration of 110 µg/mL onto SR7000 GOLD SENSOR SLIDE (catalog no. 13206066) with a signal of approximately 2600 (SENP1) and 2100 (SENP1(C603A) micro-refractive index units (µRIUs). PBST (0.5% Tween-20 and 2‰ DMSO in PBS) was used as running buffer solutions in all experiments. UA (5.85, 8.78, 13.17, 19.75, 29.63, 44.44, 66.67, and 100 µM in DMSO) and UAMMC9 (5.85, 8.78, 13.17, 19.75, 29.63, and 44.44 µM in DMSO) were injected over the slide at a flow rate of 25 µL/min with 110 s contact time and 250 s dissociation time. The background response was recorded using the reference flow channel, and the background data was subtracted from the data from each injection sample. The kinetic data were analyzed using TraceDrawer software with a 1:1 binding model.

8. Water solubility determination of UA derivatives

Sample preparation: Excess solid powder of the tested compound in sealed containers was added with 2 mL ultra-pure water, and then shaken at room temperature at 300 rpm for 24 hours to achieve dissolution equilibrium. The suspension was centrifuged at 12000 rpm for 20 min at room temperature. 500 µL supernatant was taken and diluted with 500 µL methanol to avoid precipitation of saturated solution. Three samples were carried out in parallel. HPLC method: A Waters Acquity Arc UHPLC system equipped with a Waters 2998 photodiode array (PDA) detector was used. A reversed phase ZORBAX SB-C18 (4.6 × 150 mm, 5 µm) was used for chromatographic separation. The mobile phase comprised 10% water in

acetonitrile (solvent A) and 0.02% phosphoric acid in 10% acetonitrile in water (solvent B) at a constant solvent flow rate of 1.0 mL/min.

9. Molecular docking and dynamics simulation

Docking and scoring were performed by using Schrödinger software package. LigPrep was incorporated to refine UA (ligand). Ionization states within pH=7.0±2.0 were selected for docking. SENP1 crystal structure was downloaded from protein databank (PDB ID: 2IY0, http://www.rcsb.org/). Retained A chain and the C terminal of B chain (Gly96-Gly97), deleted other residues of B chain and C chain, and restored the C603A mutation of SENP1. The protein was added hydrogen atoms and charges, removed waters by Protein Preparation Wizard module. UA was docked with SENP1 by induction-fit docking method with Gly96-Gly97 as the center and the range of flexible residues was set to 10 Å. The extended sampling method was selected for conformational search, and the residues in the range of ligand 5 Å were further optimized. Finally, the possible binding modes of UA and SENP1 were obtained, and the visual structure model was generated by Chimera software (version number: 1.13).

The molecular dynamics simulation was completed by the GROMACS software. The docking complex of UA-SENP1 was placed in a water box with a boundary of 1.0 nm, and the counterion neutralization system was added. Minimized the energy of the system before the molecular dynamic of 100 ps NVT and NPT were carried out, and the system was initially balanced. Finally, the molecular dynamics simulation of 20 ns was carried out with the setting temperature of 300 K and the step size of 2 fs. The complex structure of UA-SENP1 was extracted from the results of molecular dynamics simulation. UA was replaced by UAMMC9 and carried out the Embrace calculation, the binding modes and binding energies were obtained. The visual structure model was generated by Chimera software (version number: 1.13).

10. Animal experiments

Xenograft experiments were performed in 6-week female BALB/c athymic nude mice (Jackson Laboratory) by subcutaneously injecting 5×10^6 IGROV1 CR cells within 50% Matrigel gelatinous protein mixture (Corning). Mice were randomized to receive treatment after reached a minimum tumor volume of 150 mm^3. 4 groups of mice (6 mice per group) were treated intraperitoneally with vehicle, cisplatin (8 mg/kg/2day), UA (10 mg/kg/2days), UAMMC9 (2mg/kg/2days) and combination of UA or UAMMC9 and cisplatin (10 mg/kg/2day of UA or 2 mg/kg/2day of UAMMC9 +8 mg/kg/2 day of cisplatin) for 2 weeks. A minimum of 6 tumors per group were assessed. The tumor volume was calculated according to the formula: length x ($width^2$)/2. All animal experiments were conducted in accordance with the Institution Animal Care and Use Committee of the George Washington University for laboratory animal use and care, and all relevant ethical regulations were followed.

10. Immunohistochemistry

Immunohistochemical (IHC) staining and scoring were performed as previously described (2). The quantification of immunohistochemical staining was scored blindly at least by two independent observers. Antibodies used in IHC assay are: SENP1 (ab108981, Abcam, Cambridge, UK), JAK2, p-STAT3 (ab76315; Abcam), Bcl-xL (2764S; Cell Signaling Technology) (#3776, Cell Signaling Technologies, Danvers, MA, USA).

11. Cellular thermal shift assay and in vitro thermal shift assay

Cellular thermal shift assay was as previously described (5). Briefly, cells were pretreated with MG132 (10 µM, 1 hour) then incubated with DMSO, Mc, UA or UAMMC9 (2 µM) for 4 hours. After washing with ice-cold PBS (suppled with Protease Inhibitor Cocktail, Roche), cells were aliquoted into PCR tubes (100 µL each) and then incubated at different temperatures (from 25 to 71 °C) for 4 min. After the cells were frozen and thawed twice using liquid nitrogen, proteins were isolated from the cells after centrifugation and incubated at 70 °C for 10 min for analysis by Western blotting. *In vitro* thermal shift assays were performed by mixing purified recombinant human SENP1 protein (200 ng) with either UA (1 µM) or UAMMC9 (1µM) in binding buffer [50 mM Tris (pH 8.0), 150 mM NaCl, 10 mM $MgCl_2$, 0.5 mM DTT, 30 µg/ml bovine serum albumin (BSA), and Protease Inhibitor Cocktail, Roche] for 4 h at 25 °C and then incubated at 28, 32, 39.3, 44.9, 51.9, or 67.1 °C for 4 min. Proteins were isolated by freezing and thawing twice in liquid nitrogen and then incubated at 70 °C for 10 min prior to analysis by Western blotting

12. Statistical analysis

GraphPad Prism 5.0 software was used for statistical analysis. Data were represented as the mean ± S.D. Statistical analysis was performed using one-way ANOVA or Student's t test. $P < 0.05$ was considered significant. For Kaplan Meier survival analysis, a Log-rank (Mantel-Cox) test was used to compare each of the arms.

13. Chemical synthesis of UA derivatives

(a) General

All reagents and solvents were purchased from commercial sources without further purification. Reactions were monitored by using a UV lamp or heating coloration after treatment of 5% phosphomolybdic acid ethanol solution. Flash column chromatography was performed on 300-400 mesh silica gel. ^{1}H NMR, ^{13}C NMR, and ^{19}F NMR were recorded on an AV400 spectrometer at 400 MHz/100 MHz/376 MHz (Bruker Ltd., Faellanden, Switzerland) at 600 MHz/151 MHz/564 MHz. Chemical shifts were reported in ppm using DMSO-*d6* or $CDCl_3$ solution with TMS as internal standards. Mass spectra (ESI-MS) were performed on Agilent 1200 HPLC-6310 liquid chromatography-mass spectrometer (Agilent Ltd., Palo Alto, CA, USA). High-resolution mass spectrometry (HRMS) was performed on the AB SCIEX Triple TOF 4600 liquid chromatography-mass spectrometer (SCIEX Instruments Ltd., Canada).

(b) 3β-Acetoxy-ursolic acid (UA-1)

10 g UA (21.90 mmol, 1 eq) and 0.26 g DMAP (2.10 mmol, 0.1 eq) were dissolved in 150 mL anhydrous pyridine. 8.94 g acetic anhydride (87.58 mmol, 4 eq) was added to the mixture dropwise with stirring at room temperature. Evaporated solvent under reduced pressure after UA consumed completely, the residue was extracted by 200 mL CH_2Cl_2 and 150 mL water. The organic layer was separated, washed by 1N HCl aqueous solution and dried by anhydrous Na_2SO_4. Evaporated solvent under reduced pressure to dryness to afford 8.91 g **2a** as white solid with a yield of 81.6%. Melting point: 279.8-281.9 ℃; ESI-MS *m/z* 497.53 $[M-H]^-$; ^1H NMR (400 MHz, DMSO-*d6*) δ 11.94 (s, 1H), 5.14 (s, 1H), 4.41 (dd, J_1 = 10.8 Hz, J_2 = 4.4 Hz, 1H), 2.12 (d, *J* = 11.2 Hz, 1H), 2.01 (s, 3H), 1.99 – 1.90 (m, 1H), 1.89 – 1.74 (m, 3H), 1.68 – 1.41 (m, 10H), 1.39 – 1.22 (m, 4H), 1.06 (s, 3H), 1.01 (d, *J* = 13.2 Hz, 2H), 0.92 (s, 6H), 0.84 (m, 10H), 0.76 (s, 3H).

(c) Methoxycarbonylmethyl 3β-hydroxyurs-12-en-28-oate (UA-2)

5.23 g UA (11.45 mmol, 1 eq) was dissolved in 30 mL DMF. 1.91 g anhydrous K_2CO_3 (13.74 mmol, 1.2 eq) was added and stirred at room temperature. 1.99 g methyl bromoethyl formate was added dropwise to the mixture and stirred overnight. The reaction solution was poured into 200 mL water after UA consumed completely, the precipitate was collected as a crude product by filtration and dried in vacuum. The crude product was purified by silica gel column chromatography to afford 5.21 g **UA-2** as white foamy solid with a yield of 86.1%. Melting point: 173.3-147.9 ℃; ESI-MS *m/z* 529.35 $[M+H]^+$, 551.19$[M+Na]^+$, 527.07 $[M-H]^-$; ^1H NMR (400 MHz, $CDCl_3$) δ 5.25 (t, *J* = 3.5 Hz, 1H), 4.54 (dd, J_1 = 32.0 Hz, J_2 = 15.6 Hz, 2H), 3.73 (s, 3H), 3.21 (dd, J_1 = 10.8 Hz, J_2 = 4.8 Hz, 1H), 2.25 (d, *J* = 11.2 Hz, 1H), 2.04 (td, J_1 = 13.2 Hz, J_2 = 4.4 Hz, 1H), 1.90 (dd, J_1 = 8.8 Hz, J_2 = 3.5 Hz, 2H), 1.78 (m, 3H), 1.71 (d, *J* = 4.0 Hz, 1H), 1.64 (m, 3H), 1.49 (m, 6H), 1.32 (m, 4H), 1.11 (m, 1H), 1.08 (s, 3H), 1.04 (m, 1H), 0.99 (s, 3H), 0.94 (d, *J* = 6.1 Hz, 3H), 0.91 (s, 3H), 0.86 (d, *J* = 6.4 Hz, 3H), 0.78 (s, 3H), 0.74 (s, 33H), 0.72 (d, *J* = 14.0 Hz, 1H).

(d) 3β-Hydroxyurs-12-en-28-carboxamide (UAMMC1)

4.00 g **UA-1** (8.01 mmol, 1 eq) was dissolved in 60 mL CH_2Cl_2 and stirred in an ice bath for 0.5 h. 3.05 g oxalyl chloride (24.03 mmol, 3 eq) was added dropwise to the system, then the ice bath was removed and reacted overnight at room temperature. The solvent was removed under reduced pressure, the residue was dissolved in 25 mL CH_2Cl_2 and the solvent was removed again to obtain the acyl chloride intermediate. The intermediate was dissolved in 40 mL CH_2Cl_2 and stirred at room temperature. The ammonia was added to the sodium hydroxide solid, the ammonia gas was dried by soda lime and passed into the solution of the acyl chloride intermediate. The tail gas was absorbed by 6N HCl solution. After the reaction was completed, the reaction solution was washed with distilled water and brine, and the organic layer was dried with anhydrous Na_2SO_4. After the solvent was removed under

reduced pressure, the residue was purified by silica gel column chromatography to afford 2.84 g 3-acetoxyl intermediate. Then 0.3 g intermediate was dissolved in 15 mL mixed solvent of THF and MeOH (V:V = 3:2) and stirred at room temperature. 8 mL 4N NaOH solution was added into the solution and the reaction was monitored by TLC. After the reaction was completed, removed the solvent under reduced pressure and the residue was extracted with 30 mL distilled water and 30 mL EA. The organic layer was washed twice with 10 mL brine and dried with anhydrous $MgSO_4$. 0.23 g UAMMC1 was obtained after removing solvent and dried in vacuum with a yield of 83.7%; Melting point: 282.9-283.7 ℃; ESI-MS *m/z* 454.48 $[M-H]^-$; ^1H NMR (400 MHz, $CDCl_3$) δ 5.88 (s, 1H), 5.65 (s, 1H), 5.25 (s, 1H), 3.16 (d, *J* = 10.3 Hz, 1H), 2.01 – 1.76 (m, 6H), 1.66 (m, 2H), 1.57 (m, 3H), 1.46 (m, 5H), 1.41 – 1.32 (m, 2H), 1.31 – 1.23 (m, 2H), 1.18 (m, 1H), 1.04 (s, 3H), 1.02 (m, 1H), 0.92 (m, 4H), 0.89 (s, 3H), 0.86 (s, 3H), 0.80 (d, *J* = 10.4 Hz, 6H), 0.72 (s, 3H), 0.66 (d, *J* = 11.2 Hz, 1H).

(e) *N*-(2-Bromoethyl)-3β-hydroxyurs-12-en-28-carboxamide (UAMMC6)

The acyl chloride intermediate was prepared from 4.00 g **UA-1** (8.01 mmol, 1 eq) according to the above procedure. 2.00 g bromoethylamine hydrobromide (9.62 mmol, 1.2 eq) and 2.43 g TEA (24.06 mmol, 3 eq) were dissolved in 30 mL CH_2Cl_2 and added dropwise to the reaction mixture. After the reaction was completed, the solution was washed with 50 mL distilled water and brine, and the organic layer was dried with anhydrous $MgSO_4$. 4.64 g 3-acetoxyl intermediate was obtained after removing solvent. 0.3 g 3-acetoxyl intermediate were dissolved in 15 mL mixed solvent of THF and MeOH (V:V = 3:2) and stirred at room temperature. 8 mL 4N NaOH solution was added into the solution and the reaction was monitored by TLC. After the reaction was completed, removed the solvent under reduced pressure and the residue was extracted with 30 mL distilled water and 30 mL EA. The organic layer was washed twice with 10 mL brine and dried with anhydrous $MgSO_4$. 0.24 g **UAMMC6** was obtained after removing solvent and dried in vacuum with the yield of 86.0%; Melting point: 258.5-260.3 ℃; TOF-HRMS *m/z*: calcd for $C_{32}H_{52}BrNO_2$ 561.3181; found 482.3999 $[M-HBr]^-$; ^1H NMR (400 MHz, $CDCl_3$) δ 5.21 (t, *J* = 3.2 Hz, 1H), 4.12 (t, *J* = 9.2 Hz, 2H), 3.77 (t, *J* = 9.2 Hz, 2H), 3.21 (dd, J_1 = 10.2 Hz, J_2 = 4.0 Hz, 1H), 2.20 (d, *J* = 11.1 Hz, 1H), 2.06 (t, *J* = 13.2 Hz, 1H), 1.92 (m, 3H), 1.65 (m, 5H), 1.50 (m, 5H), 1.33 (m, 5H), 1.08 (m, 4H), 1.00 (m, 5H), 0.93 (m, 6H), 0.85 (d, *J* = 5.7 Hz, 3H), 0.77 (s, 6H), 0.72 (d, *J* = 11.2 Hz, 1H).

(f) *N*-(Methoxycarbonylmethyl)-3β-acetoxyurs-12-en-28-carboxamide (UA-3)

The acyl chloride intermediate was prepared from 4.00 g **UA-1** (8.01 mmol, 1 eq) according to the above procedure. 1.36 g glycine methyl ester hydrochloride (9.62 mmol, 1.2 eq) and 2.43 g TEA (24.06 mmol, 3 eq) were dissolved in 30 mL CH_2Cl_2 and added dropwise to the reaction mixture. After the reaction was completed, the solution was washed with 50 mL distilled water and brine, and the organic layer was dried with anhydrous

$MgSO_4$. Removed the solvent under reduced pressure and the residue was recrystallized by 15 mL MeOH. **UA-3** was white solid with a yield of 82.0%. Melting point: 115.9-117.8 ℃; ESI-MS *m/z* 570.27[M+H]$^+$, 592.49 [M+Na]$^+$, 568.34 [M-H]$^-$; ^{1}H NMR (400 MHz, $CDCl_3$) δ 6.52 (s, 1H), 5.40 (t, *J* = 3.2 Hz, 1H), 4.49 (dd, J_1 = 10.2 Hz, J_2 = 4.0 Hz, 1H), 4.10 (d, *J* = 18.8 Hz, 1H), 3.83 (d, *J* = 18.8 Hz, 1H), 3.76 (s, 3H), 2.04 (s, 3H), 1.98 (m, 3H), 1.86 (d, *J* = 13.2 Hz, 1H), 1.73 (d, *J* = 13.2 Hz, 1H), 1.60 (m, 4H), 1.48 (m, 6H), 1.31 (m, 5H), 1.09 (s, 3H), 1.03 (m, 2H), 0.95 (s, 3H), 0.93 (s, 3H), 0.89 (d, *J* = 6.0 Hz, 3H), 0.85 (s, 6H), 0.83 (d, *J* = 11.2 Hz, 1H), 0.71 (s, 3H).

(g) *N*-Methylcarbamoylmethyl 3β-hydroxyurs-12-en-28-oate (UAMMC2)

0.3 g **UA-2** (0.567 mmol, 1 eq) was dissolved in 15 mL methanol and stirring at room temperature. 10 mL 33% methylamine methanol solution was added to the mixture, and the solution gradually became turbid. The reaction was monitored by TLC. After **UA-2** was consumed completely, the reaction solution was filtered and the precipitate was washed again with 5 mL methanol. 0.23 g white powder of **UAMMC2** was obtained after drying in vacuum with a yield of 76.8%. Melting point: 246.0-247.1 ℃; ESI-MS *m/z* 550.46 [M+Na]$^+$, 526.44 [M-H]$^-$; TOF-HRMS *m/z*: calcd for $C_{33}H_{53}NO_4$ 527.3975; found 528.4036 [M+H]$^+$; ^{1}H NMR (400 MHz, $CDCl_3$) δ 6.28 (s, 1H), 5.26 (t, *J* = 3.2 Hz, 1H), 4.78 (d, *J* = 15.7 Hz, 1H), 4.24 (d, *J* = 15.7 Hz, 1H), 3.20 (dd, J_1 = 11.3 Hz, J_2 = 4.4 Hz, 1H), 2.86 (d, *J* = 4.8 Hz, 3H), 2.22 (d, *J* = 11.1 Hz, 1H), 2.01 (m, 2H), 1.86 (ddd, J_1 = 18.6 Hz, J_2 = 11.3 Hz, J_3 = 2.8 Hz, 1H), 1.71 (m, 3H), 1.61 (m, 5H), 1.52 (m, 3H), 1.45 (m, 1H), 1.34 (m, 4H), 1.12 (m, , 1H), 1.09 (s, 3H), 1.03 (m, 1H), 0.97 (s, 3H), 0.95 (d, *J* = 6.0 Hz, 3H), 0.88 (s, 3H), 0.87 (d, *J* = 6.6 Hz, 3H), 0.76 (s, 3H), 0.70 (d, *J* = 10.8 Hz, 1H), 0.67 (s, 3H); ^{13}C NMR (101 MHz, $CDCl_3$) δ 175.97, 168.21, 140.23, 125.13, 79.02, 77.48, 77.16, 76.84, 62.88, 55.25, 52.99, 48.48, 47.47, 42.35, 39.55, 39.24, 38.93, 38.84, 38.62, 37.03, 36.81, 32.91, 30.58, 28.22, 27.84, 27.23, 26.01, 24.51, 23.76, 23.29, 21.24, 18.32, 17.13, 17.01, 15.73, 15.49.

(h) *N*-Hydroxyethylcarbamoylmethyl 3β-hydroxyurs-12-en-28-oate (UAMMC4)

0.3 g **UA-2** (0.567 mmol, 1 eq) and 0.18 g ethanolamine (2.84 mmol, 5 eq) were dissolved in 25 mL methanol solution and refluxed for about 24 h. The solvent was removed under reduced pressure, and the residue was extracted with 20 mL water and 20 mL EA. The organic layer was washed twice with saturated salt and dried with anhydrous MgSO4. After the solvent was removed under reduced pressure, 0.23 g white solid powder of **UAMMC4** was obtained with the yield was 72.7%. Melting point: 121.5-123.3 ℃; ESI-MS *m/z* 556.27 [M-H]$^-$; TOF-HRMS *m/z*: calcd for $C_{34}H_{55}NO_5$ 557.4080; found 558.4153 [M+H]$^+$; ^{1}H NMR (400 MHz, $CDCl_3$) δ 6.66 (t, *J* = 5.2 Hz, 1H), 5.29 (t, *J* = 3.3 Hz, 1H), 4.71 (d, *J* = 15.8 Hz, 1H), 4.33 (d, *J* = 15.8 Hz, 1H), 3.75 (t, *J* = 5.0 Hz, 2H), 3.53 (ddd, J_1 = 15.6 Hz, J_2 = 10.6 Hz, J_3 =5.2 Hz, 1H), 3.41 (ddd, J_1 = 15.6 Hz, J_2 = 10.6 Hz, J_3 =5.2 Hz, 1H), 3.20 (dd, J_1 = 11.4 Hz, J_2 = 4.4 Hz, 1H), 2.22 (d, *J* = 11.1 Hz, 1H), 2.06 (m, 2H), 1.90 (m, 3H), 1.73 (m, 3H), 1.63 (m, 4H), 1.51 (m, 4H),

1.34 (m, 5H), 1.12 (m, 1H), 1.09 (s, 3H), 1.03 (m, 1H), 0.97 (s, 3H), 0.95 (d, *J* = 6.1 Hz, 3H), 0.89 (s, 3H), 0.86 (d, *J* = 6.4 Hz, 3H), 0.77 (s, 3H), 0.70 (d, *J* = 11.6 Hz, 1H), 0.69 (s, 3H).

(i) *N*-Hydroxyethyl-*N´*-methylcarbamoylmethyl 3β-hydroxyurs-12-en-28-oate (UAMMC5)

UAMMC5 was prepared from *N*-methylethanolamine according to the synthetic procedure of **UAMMC4**. **UAMMC5** was white solid with a yield of 55.5%. Melting point: 121.5-123.3 ℃; ESI-MS *m/z* 572.54 $[M+H]^+$, 594.28$[M+Na]^+$; TOF-HRMS *m/z*: calcd for $C_{35}H_{57}NO_5$ 571.4237; found 572.4290 $[M+H]^+$; ^1H NMR (400 MHz, $CDCl_3$) δ 5.22 (m, 1H), 4.75 and 4.64 (dd, *J* = 28.4, 14.2 Hz, 2H), 3.75 (m, 2H), 3.52 and 3.39 (t, *J* = 4.8 Hz, 1H), 3.20 (dd, J_1 = 10.8 Hz, J_2 = 4.7 Hz, 1H), 3.04 and 2.94 (s, 3H), 2.25 (d, *J* = 11.2 Hz, 1H), 2.03 (m, 1H), 1.89 (m, 2H), 1.76 (m, 4H), 1.61 (m, 3H), 1.48 (m, 5H), 1.33 (m, 4H), 1.08 (m, 4H), 1.01 (m, 1H), 0.97 (m, 4H), 0.92 (d, *J* = 6.2 Hz, 3H), 0.90 (s, 3H), 0.84 (d, *J* = 6.4 Hz, 3H), 0.76 (s, 3H), 0.73 (d, *J* = 4.4 Hz, 3H), 0.70 (d, *J* = 11.6 Hz, 1H).

(j) *N*-(*N*-Methylcarbamoylmethyl)-3β-hydroxyurs-12-en-28-carboxamide (UAMMC7)

0.6 g **UA-3** (1.052 mmol, 1 eq) was dissolved in 15 mL methanol and stirring at room temperature. 10 mL 33% methylamine methanol solution was added to the mixture, and the solution gradually became turbid. After **UA-3** was consumed completely, the reaction solution was filtered and the precipitate was washed again with 5 mL methanol. 0.43 g white powder of 3-acetoxyl intermediate was obtained after drying in vacuum. 0.3 g intermediate were dissolved in 15 mL mixed solvent of THF and MeOH (V:V = 3:2) and stirred at room temperature. 8 mL 4N NaOH solution was added into the solution and the reaction was monitored by TLC. After the reaction was completed, removed the solvent under reduced pressure and the residue was extracted with 30 mL distilled water and 30 mL EA. The organic layer was washed twice with 10 mL brine and dried with anhydrous $MgSO_4$. 0.26 g **UAMMC7** was obtained after removing solvent and dried in vacuum with the yield of 93.6%; Melting point: 237.3-238.8 ℃; ESI-MS *m/z* 527.35 $[M+H]^+$, 549.44 $[M+Na]^+$; TOF-HRMS *m/z*: calcd for $C_{33}H_{54}N_2O_3$ 526.4134; found 527.4178 $[M+H]^+$; ^1H NMR (400 MHz, $CDCl_3$) δ 6.92 (s, 1H), 5.41 (t, *J* = 3.2 Hz, 1H), 3.98 (d, *J* = 14.2 Hz, 1H), 3.79 (d, *J* = 15.6 Hz, 1H), 3.52 (brs, 1H), 3.21 (dd, J_1 = 11.2 Hz, J_2 = 4.4 Hz, 1H), 2.81 (s, 3H), 2.01 (m, 4H), 1.82 (m, 1H), 1.70 (m, 1H), 1.51 (m, 6H), 1.32 (m, 3H), 1.09 (s, 3H), 1.04 (m, 1H), 0.99 (m, 1H), 0.98 (s, 3H), 0.95 (s, 3H), 0.90 (s, 3H), 0.87 (d, *J* = 6.4 Hz, 3H), 0.77 (s, 3H), 0.71 (d, *J* = 11.2 Hz, 1H), 0.69 (s, 3H).

2.11

(k)*N*-[*N*-(2-Hydroxyethyl)-*N*-methyl-carbamoylmethyl]-3β-hydroxyurs-12-en-28-carbox-amide (UAMMC9)

0.3 g **UA-3** (0.526 mmol, 1 eq) and 0.4 g *N*-methyl ethanolamine (5.26 mmol, 10 eq) were dissolved in 25 mL methanol and heated to reflux. The reaction was monitored by TLC, the solvent was removed under reduced pressure after the reaction was completed. The

residue was extracted with 30 mL distilled water and 30 mL EA. The organic layer was washed twice with 30 mL brine and dried with anhydrous $MgSO_4$. After the solvent was removed under reduced pressure, the crude products of the corresponding intermediates were obtained, which were directly used in the next step without purification. The crude product was dissolved in 15 mL mixed solvent of THF and MeOH (V:V = 3:2) and stirred at room temperature. 10 mL of 4N NaOH solution was added and monitored the reaction by TLC. After the reaction was completed, evaporated solvent under reduced pressure and the residues were extracted with 30 mL distilled water and 30 mL EA. The organic layer was washed twice with 30 mL brine and dried with anhydrous $MgSO_4$. After the solvent was removed under reduced pressure, the residue was purified on a silica gel column and UAMMC9 was obtained as white solid powder with the yield of 74.2% (calculated by two steps); Melting point: 250.1-251.9 ℃; ESI-MS *m/z* 571.46 $[M+H]^+$, 593.51 $[M+Na]^+$; TOF-HRMS *m/z*: calcd for $C_{35}H_{58}N_2O_4$ 570.4397; found 571.4458 $[M+H]^+$; ^1H NMR (400 MHz, $CDCl_3$) δ 7.09 (m, 1H), 5.45 (t, J = 3.4 Hz, 1H), 4.20 and 4.07 (dd, J_1 = 17.6 Hz, J_2 = 4.4 Hz, 1H), 3.91 (dd, J_1 = 17.8 Hz, J_2 = 2.8 Hz, 1H), 3.79 and 3.76 (t, J = 5.2 Hz, 2H), 3.58 and 3.41 (ddt, J_1 = 36.0 Hz, J_2 = 14.4 Hz, J_3 =5.2 Hz, 2H), 3.21 (dd, J_1 = 11.2 Hz, J_2 = 4.4 Hz, 1H), 3.04 and 3.00 (s, 3H), 2.63 (brs, 1H), 2.02 (m, 2H), 1.95 (m, 2H), 1.79 (m, 4H), 1.63 (m, 4H), 1.46 (m, 7H), 1.29 (m, 4H), 1.09 (s, 3H), 1.04 (m, 2H), 0.97 (s, 3H), 0.94 (m, 3H), 0.89 (s, 3H), 0.87 (d, J = 7.4 Hz, 3H), 0.76 (s, 3H), 0.71 (d, J = 11.6 Hz, 1H), 0.67 (s, 3H).

Nuclear magnetic resonance spectrums

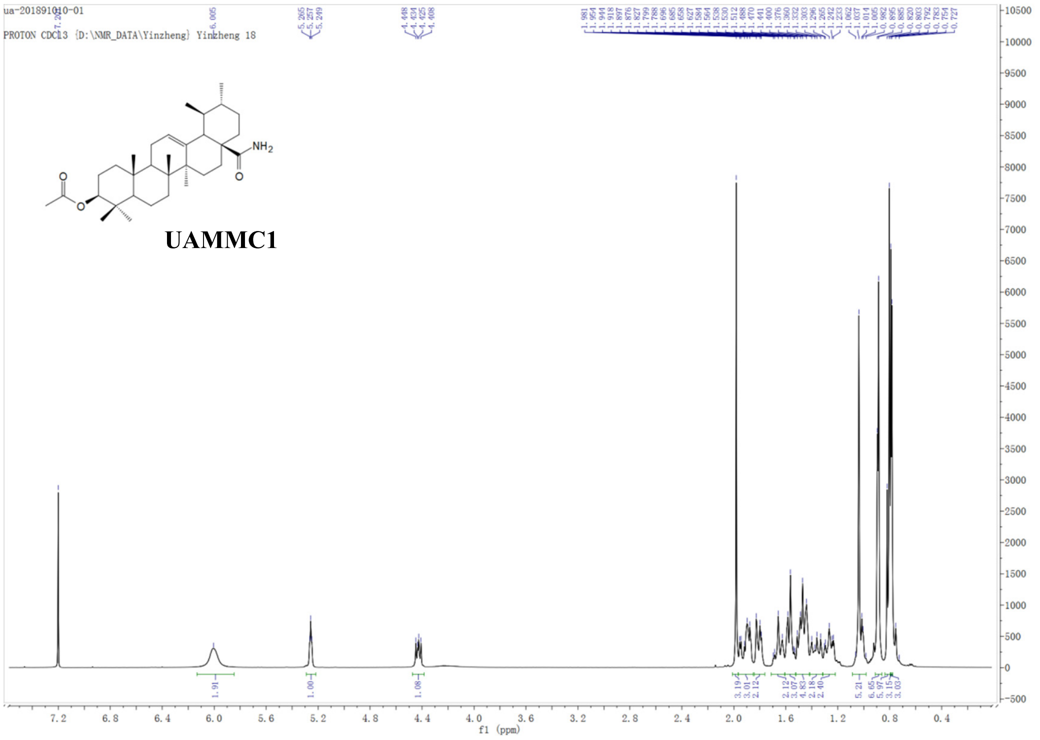

^{1}H NMR spectrum of **UAMMC1**

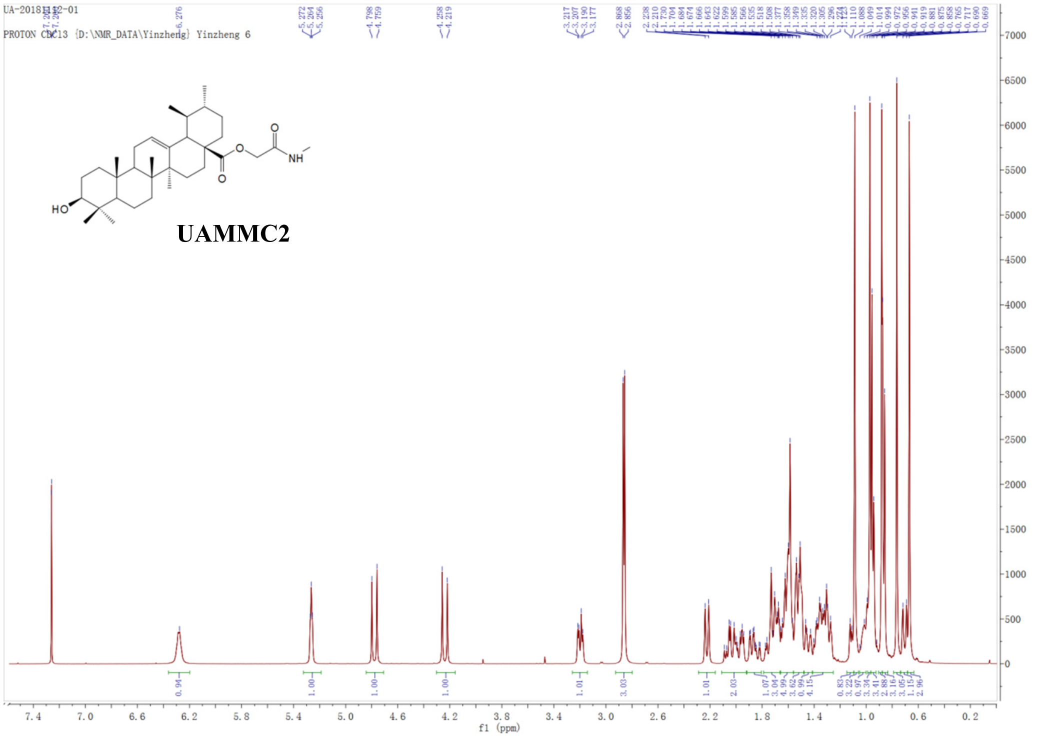

^{1}H NMR spectrum of **UAMMC2**

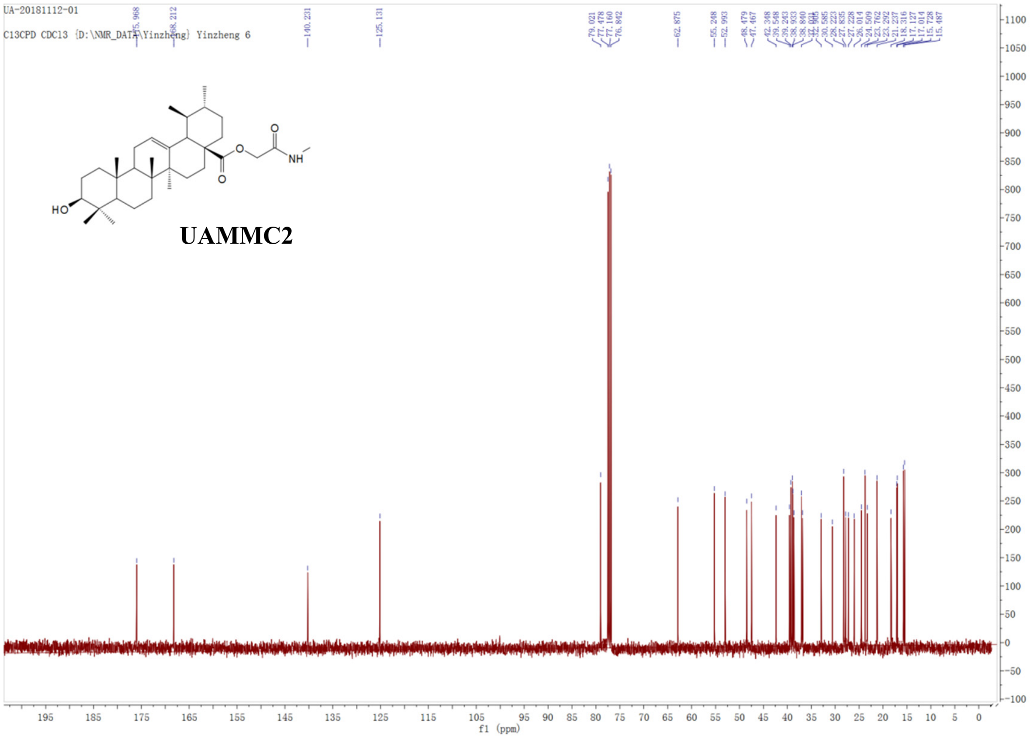

^{13}C NMR spectrum of **UAMMC2**

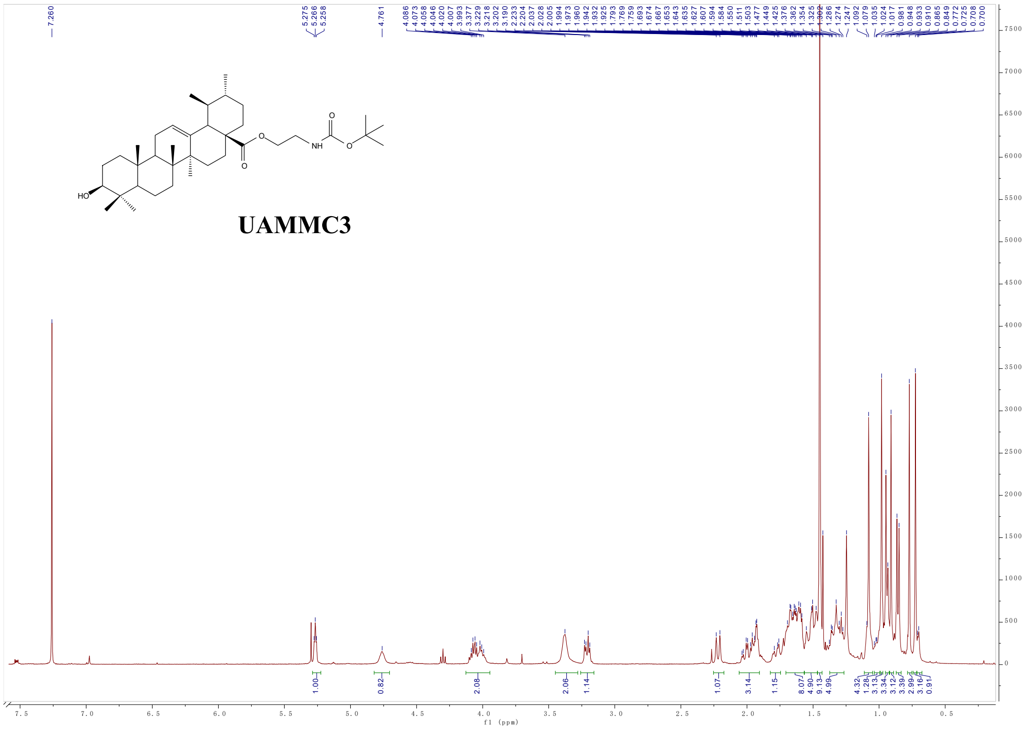

^{1}H NMR spectrum of **UAMMC3**

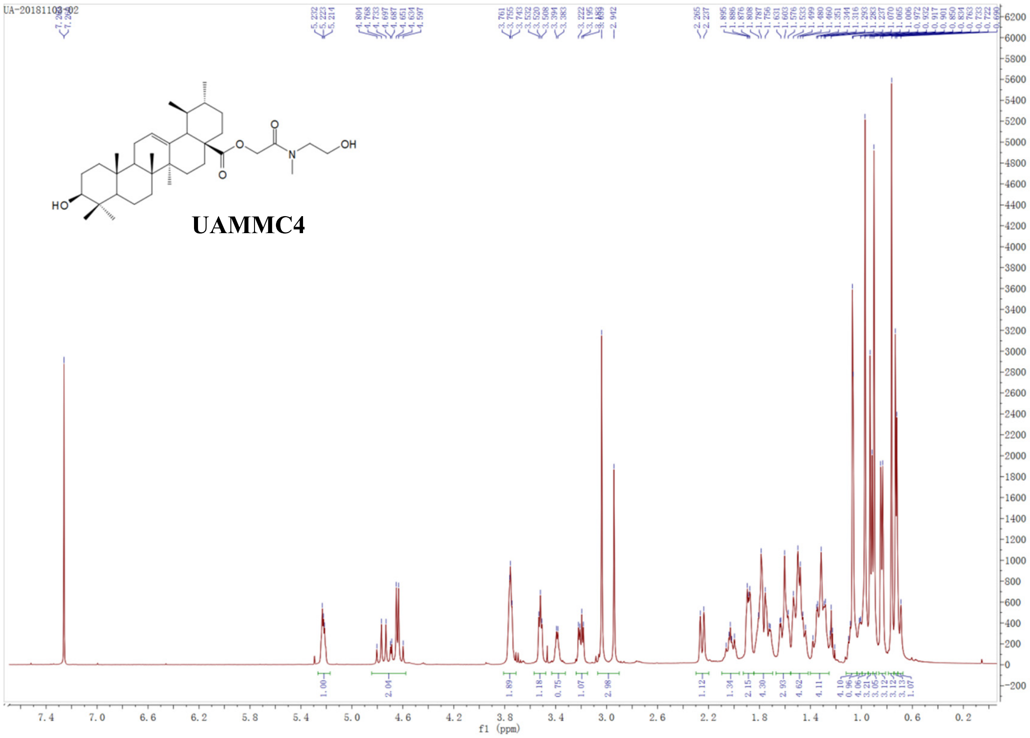

^{1}H NMR spectrum of **UAMMC4**

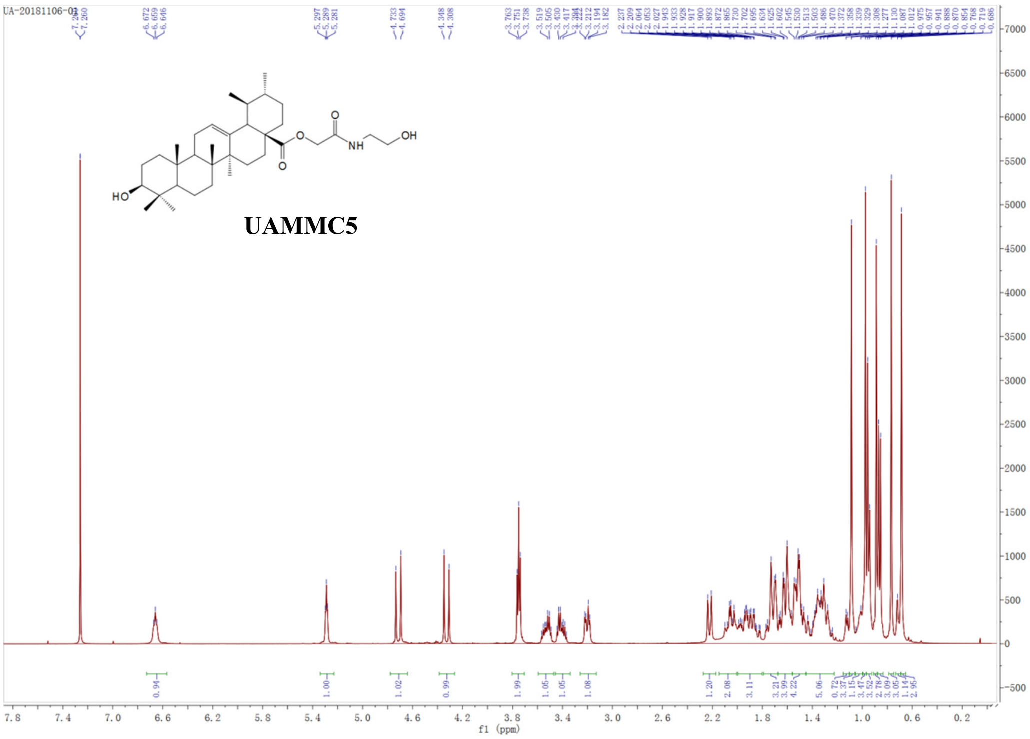

^{1}H NMR spectrum of **UAMMC5**

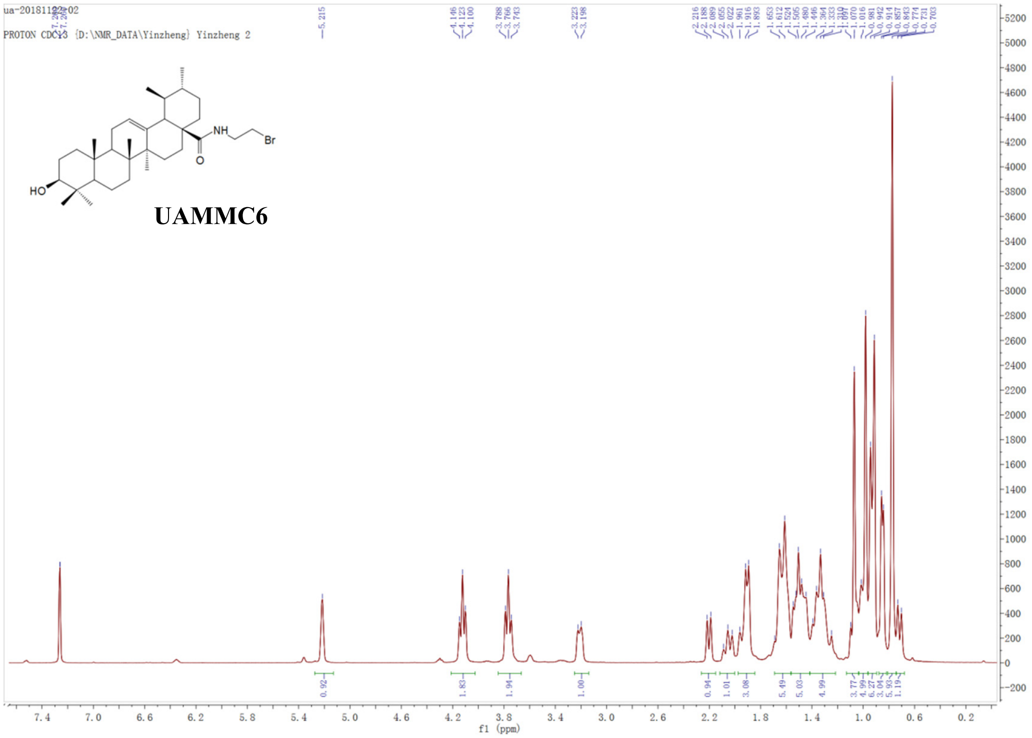

^{1}H NMR spectrum of **UAMMC6**

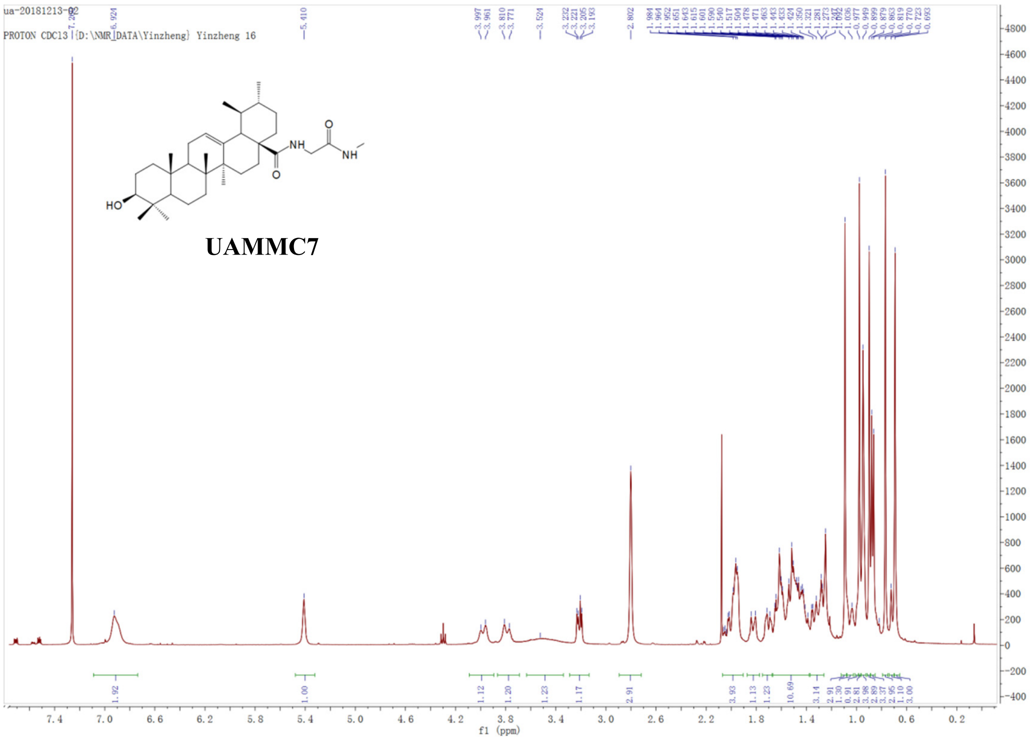

^{1}H NMR spectrum of **UAMMC7**

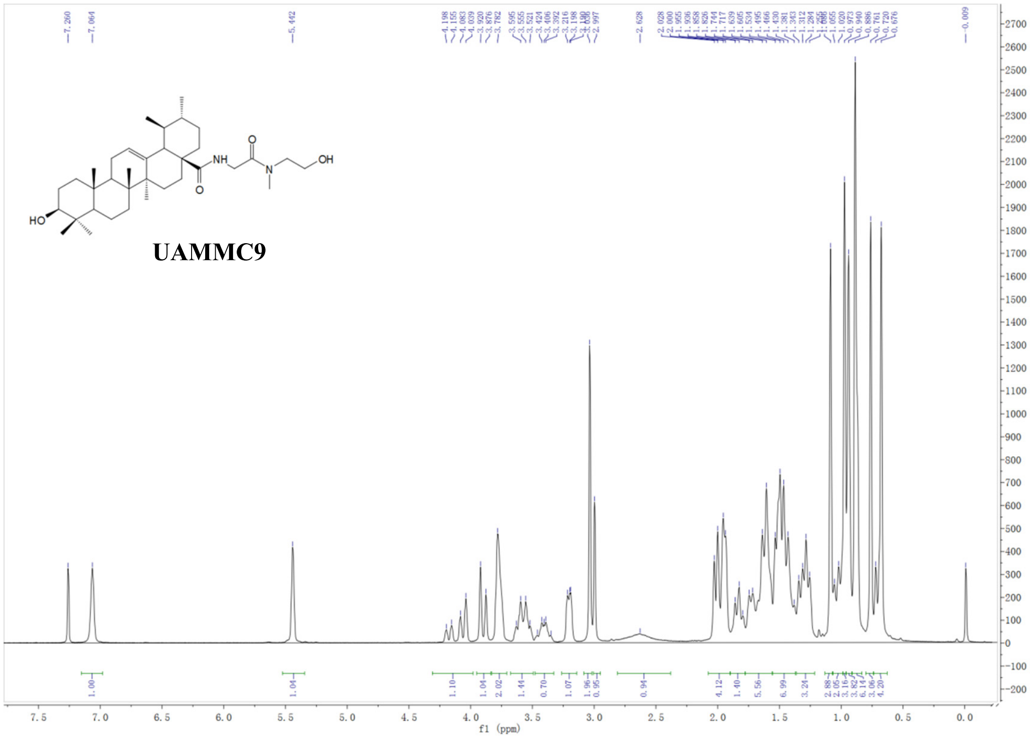

^{1}H NMR spectrum of **UAMMC9**

Mass spectrometry

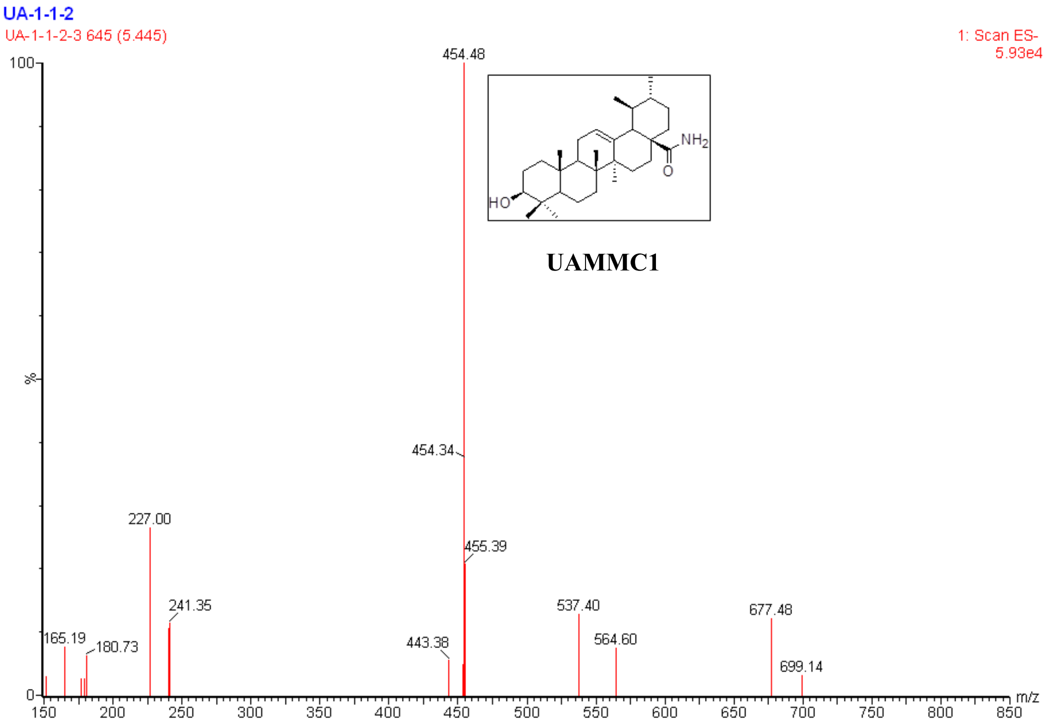

ESI-Mass spectrometry of **UAMMC1**

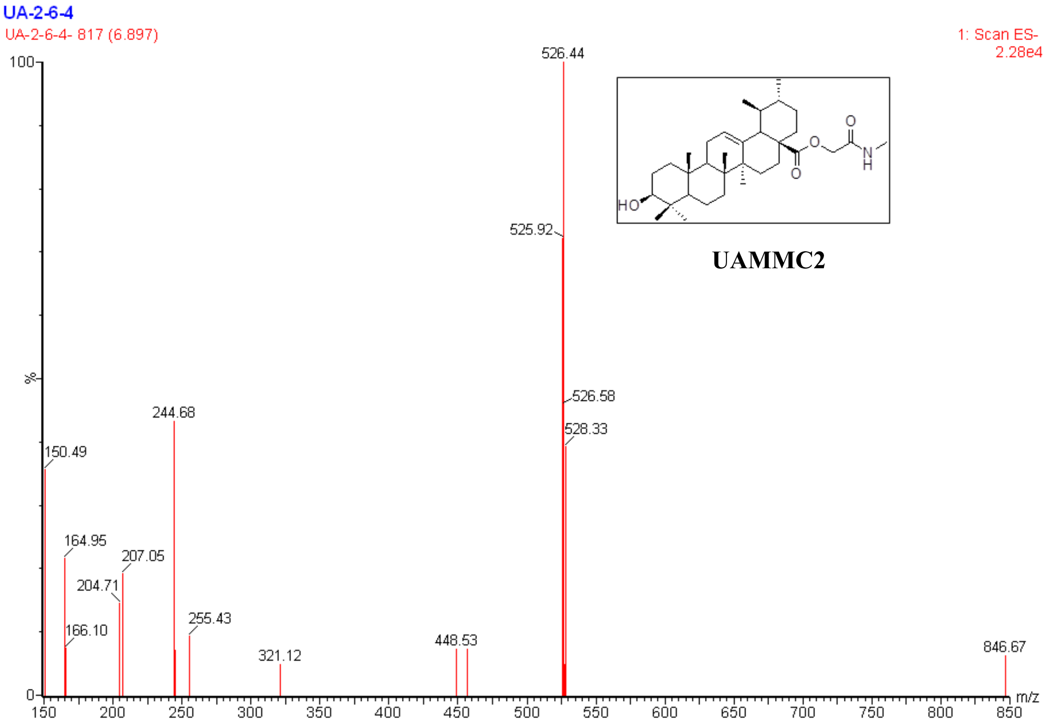

ESI-Mass spectrometry of **UAMMC2**

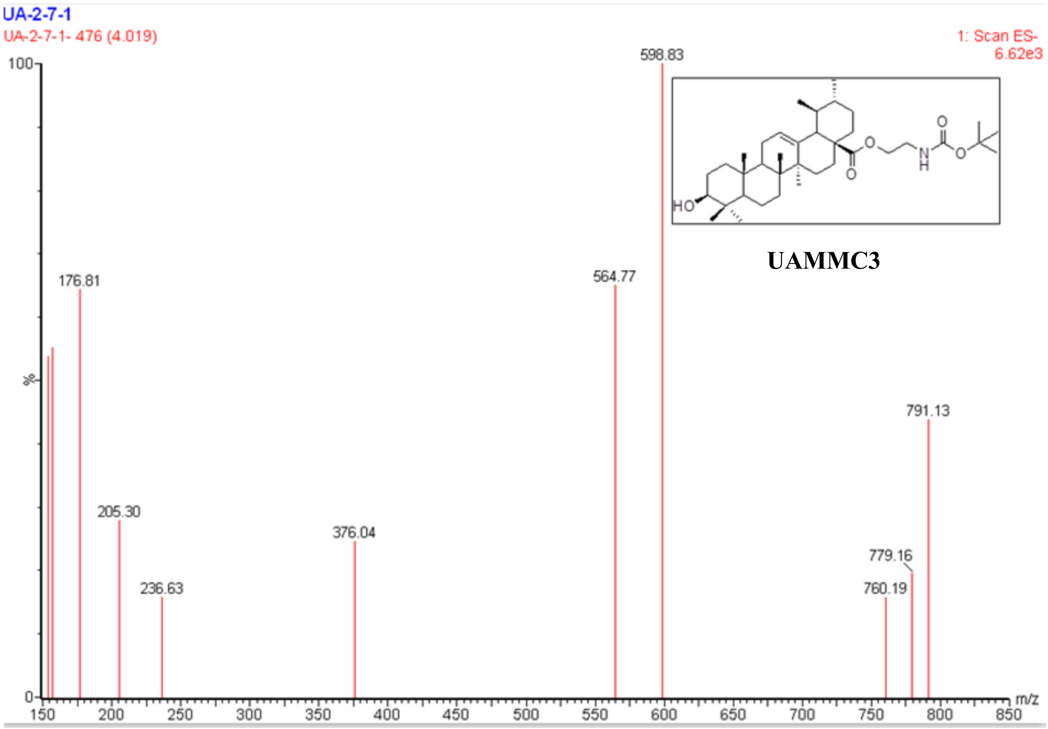

ESI-Mass spectrometry of **UAMMC3**

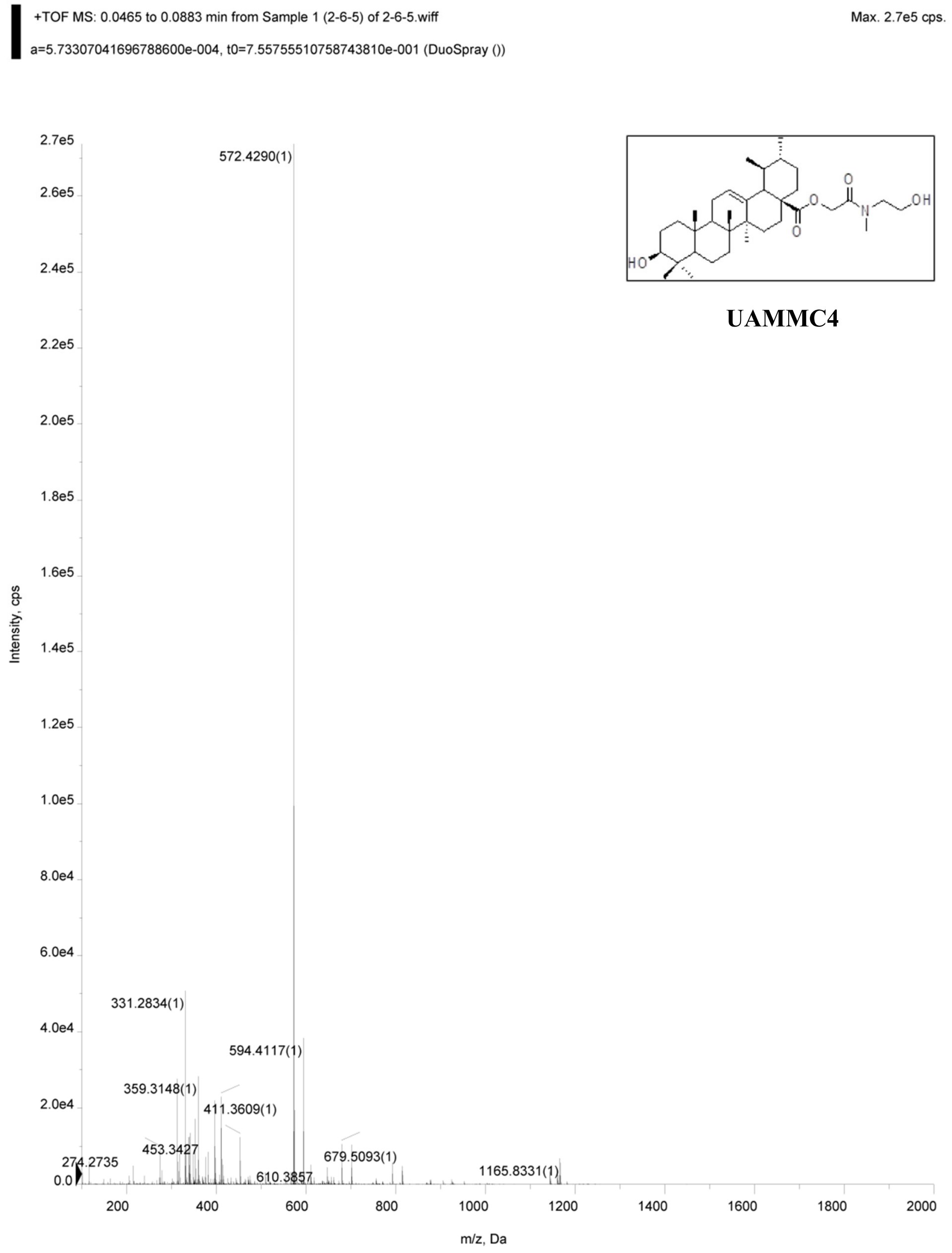

High-resolution mass spectrometry of **UAMMC4**

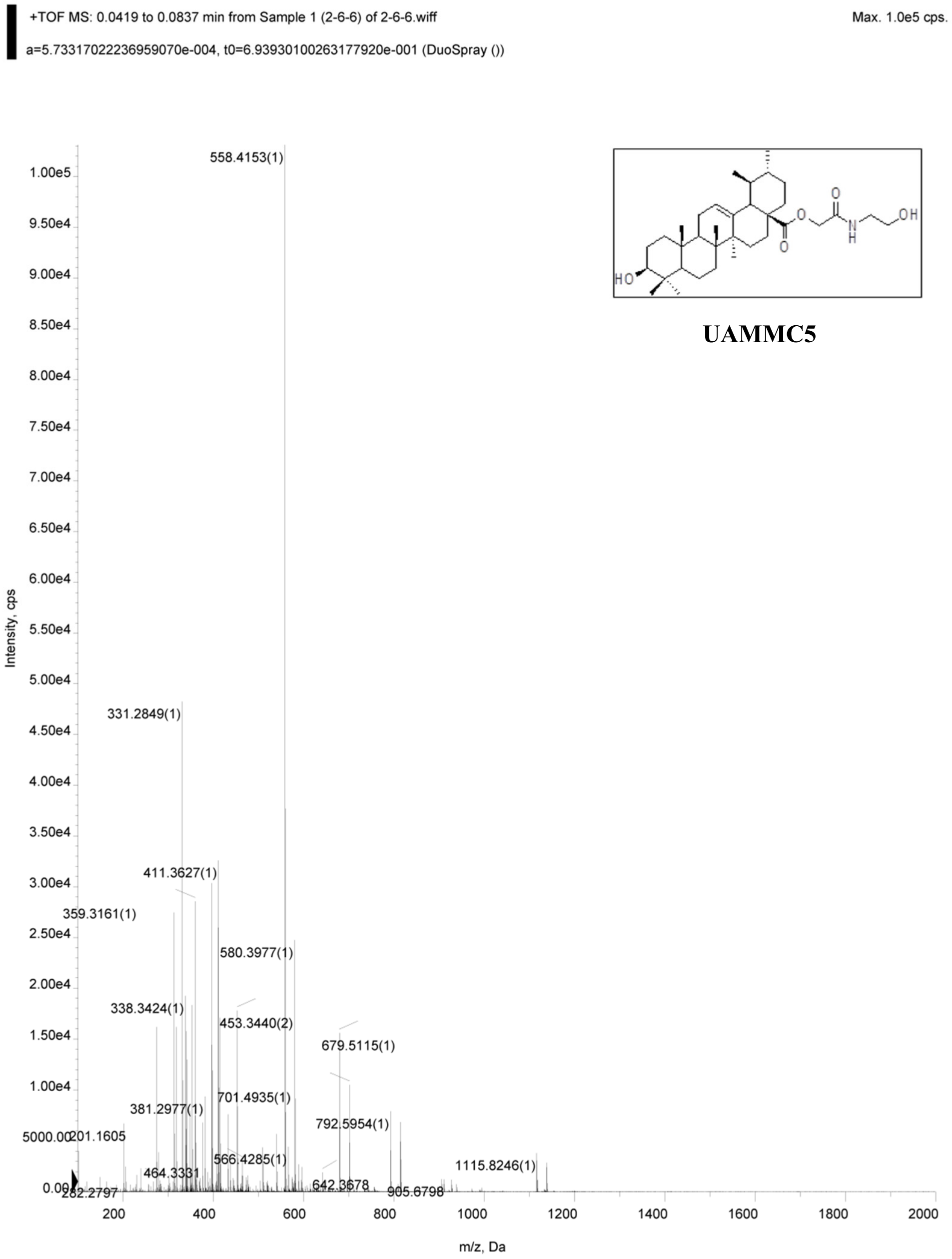

High-resolution mass spectrometry of **UAMMC5**

7c

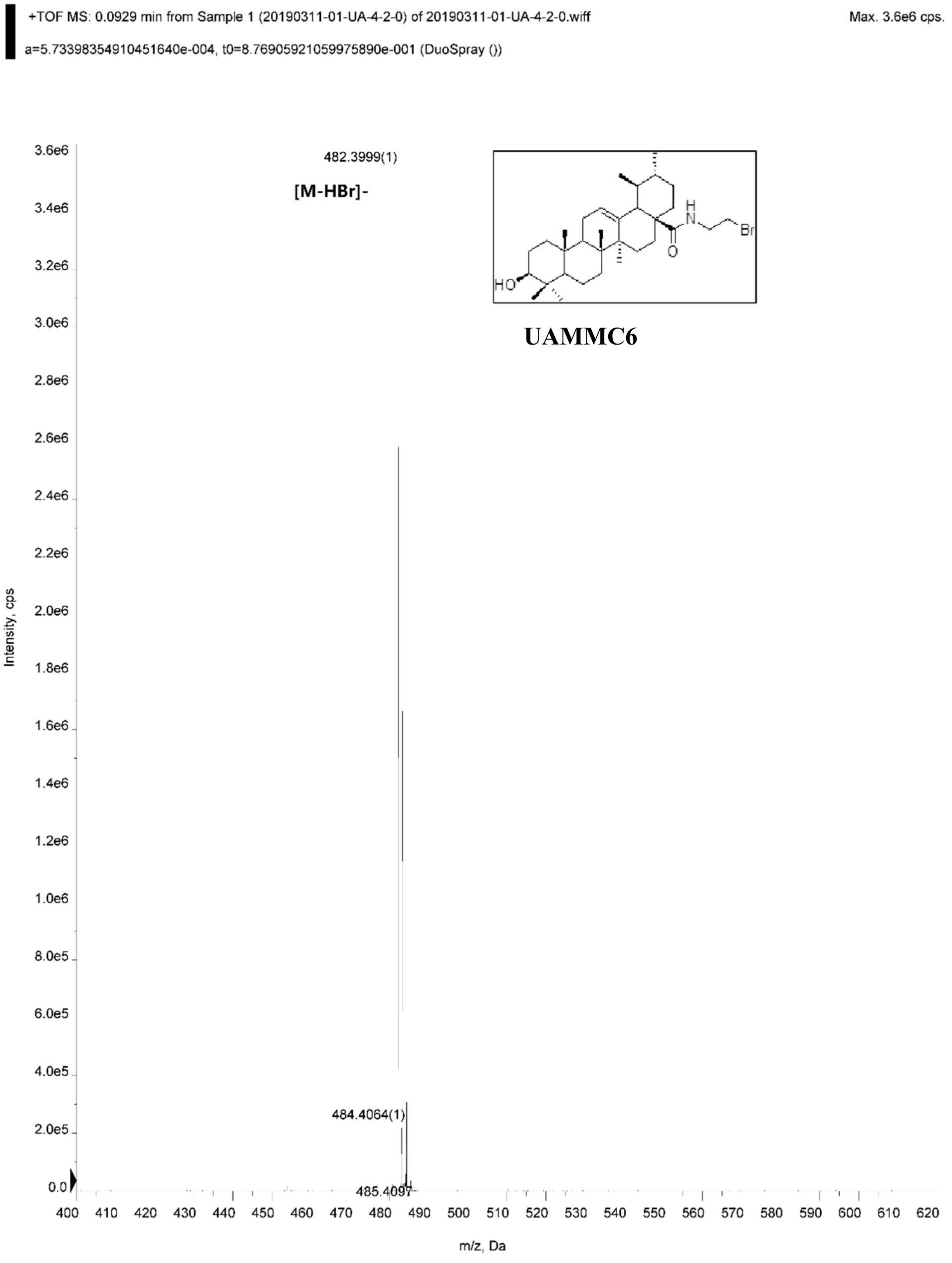

High-resolution mass spectrometry of **UAMMC6**

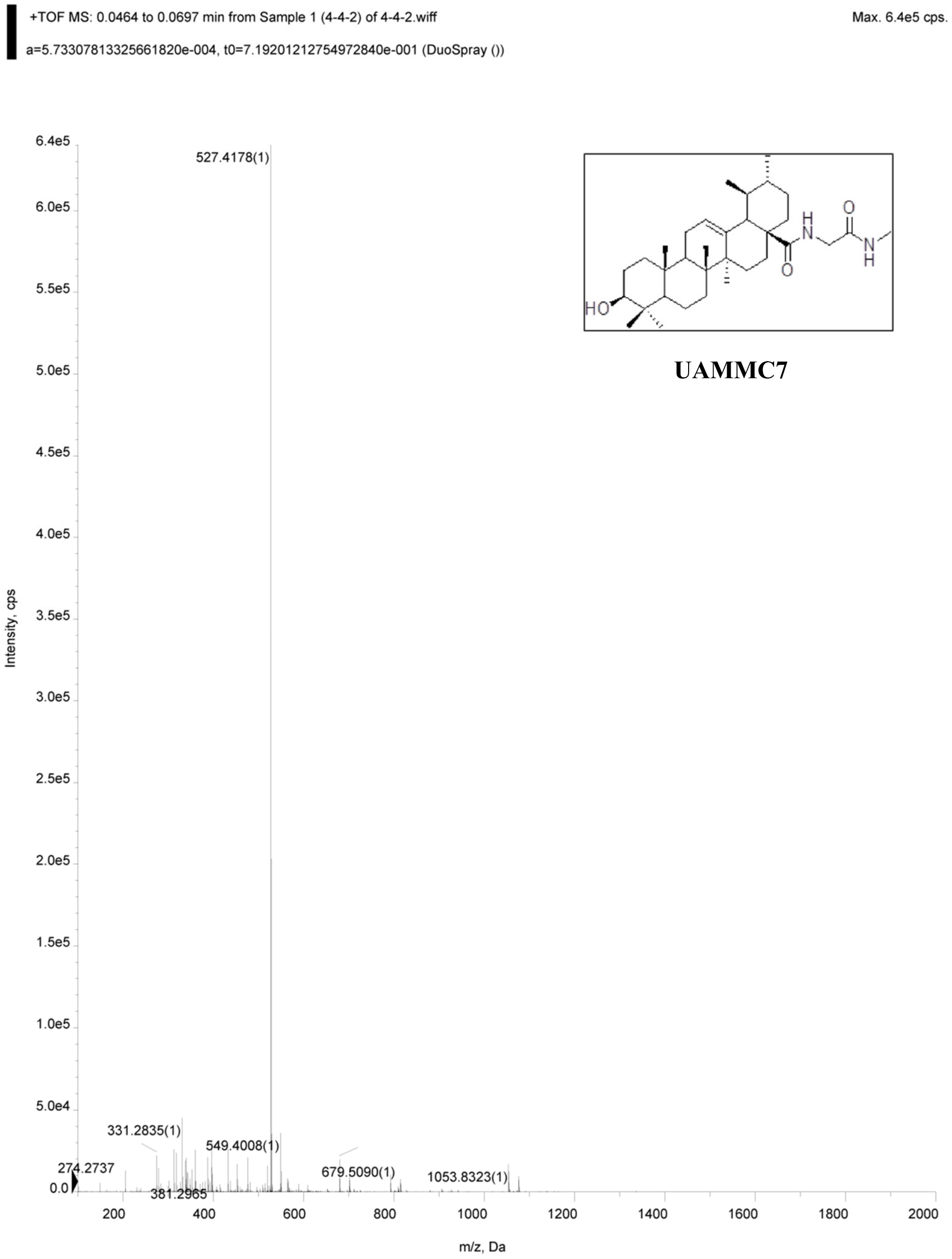

High-resolution mass spectrometry of **UAMMC7**

+TOF MS: 0.0371 to 0.0976 min from Sample 1 (4-4-5) of 4-4-5.wiff Max. 3.3e5 cps.

a=5.73322568306360110e-004, t0=8.10962978925395260e-001 (DuoSpray ())

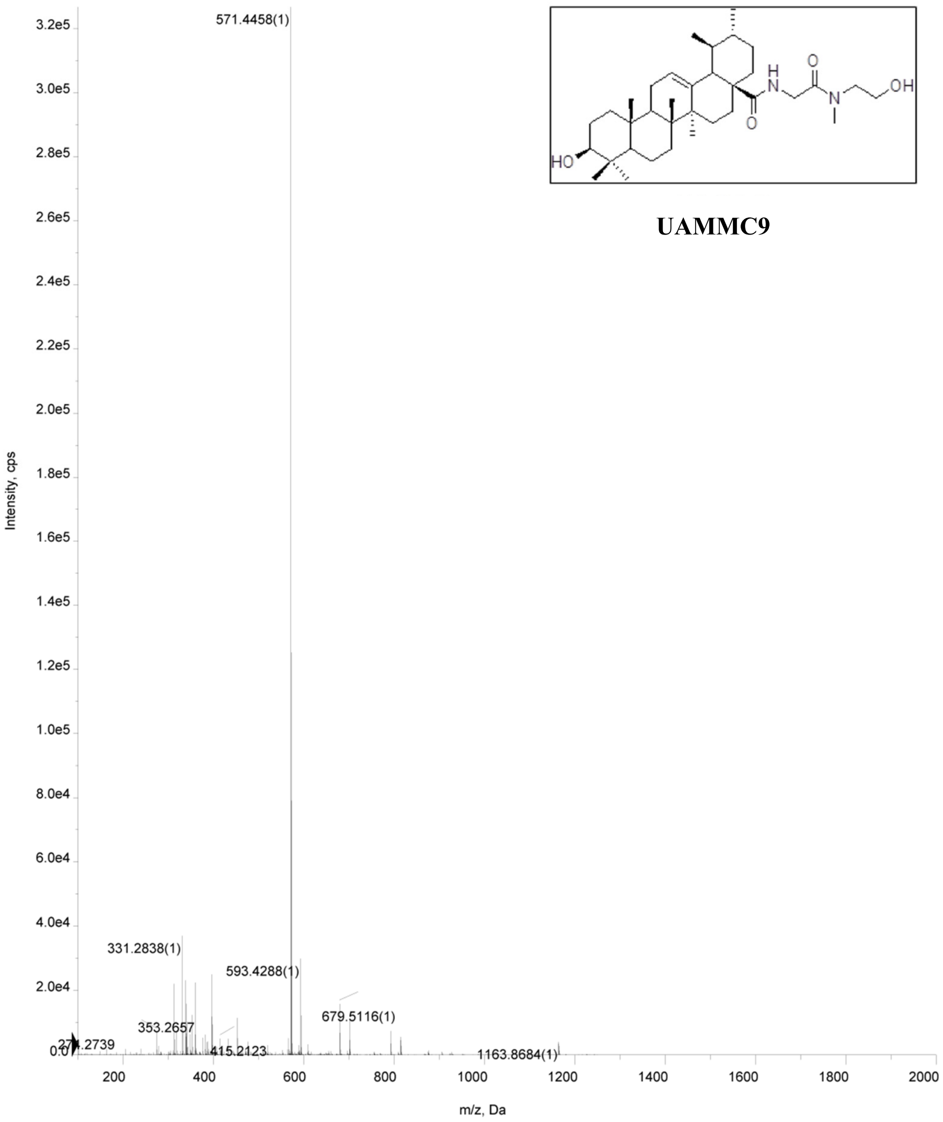

UAMMC9

High-resolution mass spectrometry of **UAMMC9**

References

1. Li J, Wu R, Yung MMH, Sun J, Li Z, Yang H*, et al.* SENP1-mediated deSUMOylation of JAK2 regulates its kinase activity and platinum drug resistance. Cell Death Dis **2021**;12:341
2. Zhou W, Sun W, Yung MMH, Dai S, Cai YH, Chen CW*, et al.* Autocrine activation of JAK2 by IL-11 promotes platinum drug resistance. Oncogene **2018**;37:3981-97
3. Vichai V, Kirtikara K. Sulforhodamine B colorimetric assay for cytotoxicity screening. Nature protocols **2006**;1:1112-6
4. Chou TC, Talalay P. Quantitative analysis of dose-effect relationships: the combined effects of multiple drugs or enzyme inhibitors. Advances in enzyme regulation **1984**;22:27-55
5. Chen CW, Li Y, Hu S, Zhou W, Meng Y, Li Z*, et al.* DHS (trans-4,4'-dihydroxystilbene) suppresses DNA replication and tumor growth by inhibiting RRM2 (ribonucleotide reductase regulatory subunit M2). Oncogene **2019**;38:2364-79
